# *In silico* analysis of sarcomere length effects on myocardial contraction and cardiac function using the living left heart model

**DOI:** 10.3389/fbioe.2026.1756525

**Published:** 2026-04-10

**Authors:** Tae-Rim Kim, Chiseung Lee

**Affiliations:** 1 Department of Biomedical Engineering, Graduate School, Pusan National University, Busan, Republic of Korea; 2 Department of Biomedical Engineering, School of Medicine, Pusan National University, Busan, Republic of Korea; 3 In Silico Medicine Lab, Biomedical Research Institute, Pusan National University Hospital, Busan, Republic of Korea

**Keywords:** cardiac function analysis, *in silico* analysis, living heart model, myocardial contraction simulation, sarcomere length, sarcomere mechanics

## Abstract

The cardiac sarcomere length is a major determinant of myocardial contractility, influencing the length–tension relationship, calcium sensitivity, and preload dependence. In time-varying-elastance-based constitutive myocardial models, two fundamental sarcomere-length parameters are the unloaded sarcomere length, $L_{r}$ and the zero active tension length, $L_{0}$ . These parameters regulate calcium sensitivity and contraction duration, thereby shaping myocardial mechanical behavior. Although alterations in these parameters are implicated in sarcomere-related cardiomyopathies and have been widely studied for their effects on myocardial tension generation, their multiscale consequences—from tissue-level tension generation to organ-level pump performance—have not been systematically characterized. Motivated by this gap, we investigated how variations in $L_{r}$ and $L_{0}$ , individually and in combination, would modify global and regional left heart mechanics. The Living Left Heart Model implemented in Abaqus was used to simulate three scenarios: (1) $L_{r}$ variation from 1.75 to 1.95 µm with $L_{0}$ fixed at 1.58 µm, (2) $L_{0}$ variation from 1.48 to 1.68 µm with $L_{r}$ fixed at 1.85 µm, and (3) simultaneous variation of $L_{r}$ and $L_{0}$ with their difference kept constant. Analyses were performed from the tissue scale—evaluating length–tension relationships and active stress evolution—to the organ scale, assessing fiber stretch, fiber active stress, regional strain patterns, and overall cardiac function such as pressure–volume loops. Variation in $L_{r}$ primarily modulated contractility: at the tissue level, $L_{r}$ influenced the duration of active tension, and at the organ level, increased $L_{r}$ enhanced ventricular systolic performance whereas decreased $L_{r}$ impaired it, reflecting changes in inotropy. Changes in $L_{0}$ were even more pronounced; at the tissue level, $L_{0}$ shifted peak active stress, and at the organ level, it induced diastolic dysfunction-like behavior with altered filling and relaxation. We further confirmed that the sarcomere length difference (SLD, $L_{r}-L_{0}$ ) determined the peak active stress, and that distinct parameter sets with the same SLD often produced similar organ–level responses. Collectively, these findings identify $L_{r}$ and $L_{0}$ as mechanistic control axes that shape a continuum of left heart functional phenotypes, rather than mapping one-to-one onto a single disease entity. This framework suggests that sarcomere-level parameters can be used to systematically explore and emulate sarcomere-related cardiac pathophysiology *in silico*.

## 1 Introduction

Cardiac contraction begins at the level of individual cardiomyocytes, where mechanical regulation occurs at the sarcomere scale. In particular, sarcomere length (SL) plays a direct role in modulating contractile force. Changes in SL influence the development of active tension through multiple mechanisms, including alterations in myosin–actin overlap, calcium sensitivity ( ${Ca}^{2+}$ sensitivity), and contraction duration (Crocini and Gotthardt, 2021; Schmidt et al., 2021). Accordingly, numerous studies have investigated the relationship between cardiac sarcomere length and both active and passive tension. In particular, mutations in genes encoding titin proteins, such as RBM20, have been shown to alter sarcomere length, thereby modulating ${Ca}^{2+}$ sensitivity and affecting passive tension (de Tombe and ter Keurs, 2016; Li et al., 2017). Arts et al. also reported that mutations in the RBM20 gene increase the compliance of titin, resulting in further elongation of sarcomeres during relaxation (Arts et al., 2024). This elongation reduces active tension during contraction, consequently decreasing myocardial contractility and increasing end-diastolic pressure. Reflecting these findings, the time-varying elastance (TVE) model was developed to incorporate SL as one of its variables for calculating time-dependent active stress. This model, originally proposed by Guccione et al., computes active fiber stress over time by considering ${Ca}^{2+}$ concentration, the availability of actin-binding sites, and sarcomere length (Guccione et al., 1995; Guccione et al., 2001). The formulation has successfully reproduced experimental observations of cardiac muscle mechanics, as demonstrated in the studies by van Heuningen et al. (1982), Krueger and Tsujioka (1986). Furthermore, Walker et al. validated the performance of a nonlinear, anisotropic 3D finite element model of ovine myocardial infarction using tagged magnetic resonance imaging, in which the TVE formulation was implemented (Walker et al., 2005).

SL, however, is not represented by a single value. In addition to $L_{r}$ —the sarcomere length in the unloaded configuration and the initial input parameter for the model—another important independent parameter is $L_{0}$ , the sarcomere length at which no active tension develops (Wisneski et al., 2020; Wisneski et al., 2022). The TVE model includes both $L_{r}$ and $L_{0}$ as key parameters. $L_{r}$ is associated with both the magnitude of active tension and the duration of contraction. In contrast, $L_{0}$ adjusts the Ca^2+^ sensitivity parameter, meaning that changes in $L_{0}$ alter the shape of the length–tension relationship curve, which in turn may significantly affect systolic behavior of the heart (Shiels and White, 2008). Nonetheless, to date, few studies have considered variations in $L_{0}$ , likely because this parameter is difficult to manipulate experimentally and is conceptually treated as a boundary condition. However, based on the experimental work by ter Keurs et al., the TVE model adopted a value of $L_{0}=1.58µm$ derived from 12 cardiac trabeculae under specific conditions (ter Keurs et al., 1980). This value continues to be used in the finite element-based Living Heart Model, developed and continuously updated by Dassault Systèmes. Recognizing this, we identified the need to study SL with both $L_{r}$ and $L_{0}$ together, which serves as the starting point for this study.

The Living Heart Model is part of the Living Heart Human Project developed by Dassault Systèmes and implemented in Abaqus (Rhode Island). The project provides computational cardiac models, including the Living Heart Human Model (LHHM), a four-chamber model comprising both ventricles and atria, and the Living Left Heart Model (LHM), which consists of the left ventricle (LV) and left atrium (LA). We use the model names and abbreviations as defined in the project documentation. To avoid confusion between the general term “Living Heart Model” and the abbreviation “LHM,” we use “Living Heart Model” in full throughout the manuscript and do not introduce an abbreviation for it. In addition to the chambers, these models incorporate associated vasculature and other anatomical structures. These models serve as valuable tools for detailed examination of heartbeat behavior from both electrical and mechanical perspectives, especially in relation to action potential dynamics (Genet et al., 2016; Costabal et al., 2019; Song et al., 2022). Cardiovascular disease remains the leading cause of death worldwide, thereby driving the increasing attention toward digital twin and computational simulation technologies for better understanding and advanced treatment development. A representative example is the Living Heart Model, developed by Dassault Systèmes since 2013 (Baillargeon et al., 2014; Reza et al., 2025).

Therefore, this study aims to achieve a deeper understanding of SL by utilizing LHM. In particular, TVE model is implemented in a finite element framework to analyze the changes in fiber active stress and stretch. The default value of $L_{r}$ in the LHM is set to 1.85 µm, based on experimental data from Rodriguez et al., which reported sarcomere lengths of 1.78 µm in the endocardium and 1.91 µm in the epicardium (Rodriguez et al., 1993). Guccione et al. also supported a global LV average SL of 1.845 µm. As for $L_{0}$ , the value of 1.58 µm has been consistently adopted in most studies following the experimental work of ter Keurs et al., and no further updates have been reported, making it the standard baseline (Guccione and McCulloch, 1993; Guccione et al., 1993). Based on these default values, we systematically vary $L_{r}$ and $L_{0}$ to examine how their variations affect not only the active stress at the myocardial tissue level but also the overall contractile behavior of the organ level, i.e., the left heart. Since $L_{r}$ has already been extensively investigated through both experimental and theoretical studies at the single sarcomere and tissue levels, its influence on active stress can serve as a control in this study. Nevertheless, conducting an in-depth investigation into the variation of $L_{r}$ is important because information on its role in the comprehensive analysis of the heart’s global mechanical behavior is scarce. Through this study, variations in $L_{r}$ enabled the examination of the spatial distribution of fiber active stress and stretch across the entire left heart at both end-diastole (ED) and end-systole (ES), as well as the analysis of time-dependent changes in pressure and the pressure–volume (PV) loop. Such analyses provide macroscopic insights into contractile behavior of the left heart that go beyond the myocardial tissue level, offering data not easily accessible in previous studies. In addition, by varying $L_{0}$ beyond the fixed value derived from ter Keurs’s experiment on 12 cardiac trabeculae (ter Keurs et al., 1980) we observed meaningful changes that may critically contribute to pathological conditions such as diastolic dysfunction.

In this study, based on the length–active tension relationship of cardiac sarcomeres, $L_{r}$ was varied within ±0.1 µm from the baseline value of 1.85 µm, and $L_{r}$ was similarly varied within ±0.1 µm from the reference value of 1.58 µm. Through three scenarios—modifying $L_{r}$ alone, $L_{0}$ alone, and both together—we assessed how variations in sarcomere length parameters influence mechanical contraction behavior as reflected by regional deformation quantified using average peak strain (APS) and cardiac performance of the left heart, expressed through functional metrics such as stroke volume (SV), left ventricular ejection fraction (LVEF) and cardiac output (CO). We also identified potential links to specific heart disease conditions.

## 2 Materials and methods

### 2.1 Living left heart model (LHM)

LHM used in this study is a two-chamber left-heart finite-element model consisting of the LV and LA, generated from medical imaging of a healthy adult male heart. We selected this model because it provides substantially shorter computation times than LHHM while retaining the key left-heart mechanics required for the present parametric study. Mechanical simulations were performed in Abaqus/Explicit. Chamber pressures were not prescribed *a priori*; instead, they emerged from coupling the deforming LV and LA cavities to the default closed-loop lumped-parameter circulation through fluid-cavity formulations and resistive flow links. Specifically, instantaneous chamber pressures were computed from the time-varying cavity volumes through the compliance relations of the closed-loop lumped-parameter model, so myocardial contraction modulates pressures by altering cavity volume. Blood flow between compartments was governed by valve-like one-directional resistive elements, such that transvalvular pressure differences drove forward flow and determined sequential valve opening and closing. Model initialization followed the standard preload procedure. During the preload step, hydrostatic cavity pressures were ramped from zero to the target preload pressures reported for normal conditions at 70% diastole while the tissue reached a physiologic pre-stressed state. At the start of the beat step, the prescribed preload pressure boundary conditions were removed, and subsequent cycles proceeded under the default closed-loop circulation with a constant overall blood volume. Myocardial activation was driven by the LHM built-in action-potential–based electrical activation rather than a spatially uniform contraction. Ventricular material orientations were assigned element-wise using the LHM default rule-based fiber algorithm, with a prescribed transmural helix-angle variation from approximately −60° at the epicardium to +60° at the endocardium; papillary muscle fibers were defined to be parallel to their long axes. The same fiber field and default circulatory, electrical, and structural settings were used for all simulation cases so that differences in outcomes reflect only the parameters varied in the scenario design.

### 2.2 The cardiac material models

The passive myocardial tissue behavior in the Living Heart Model (LHM/LHHM) is described by the anisotropic hyperelastic formulation proposed by Holzapfel and Ogden (Holzapfel and Ogden, 2009). The strain energy density is decomposed into deviatoric and volumetric parts, as given in Equation 1. The deviatoric contribution is further written as the sum of an isotropic matrix term, transversely isotropic fiber/sheet reinforcement terms, and a fiber -sheet coupling term, as defined in Equations 2-5

$$\psi =\psi_{dev}^{i}+\psi_{dev}^{t}+\psi_{dev}^{o},$$ (1)

$$\psi_{dev}^{i}=\frac{a}{2b}\exp\left[b\left(I_{1}-3\right)\right],$$ (2)

$$\psi_{dev}^{t}=\sum_{i=f,s}\frac{a_{i}}{{2b}_{i}}\left\{\exp\left[b_{i}{\left(I_{4i}-1\right)}^{2}\right]-1\right\},$$ (3)

$$\psi_{dev}^{o}=\frac{a_{fs}}{{2b}_{fs}}\left[\exp\left(b_{fs}I_{8fs}^{2}\right)-1\right],$$ (4)

$$\psi_{vol}=\frac{1}{D}\left(\frac{J^{2}-1}{2}-\ln J\right),$$ (5)

where, **F** is the deformation gradient and $J=\det\left(F\right)$ . The right Cauchy-Green tensor is $C=F^{T}F,$ and $\bar{C}=J^{-2/3}C$ denotes its deviatoric part. The parameter a is related to the isotropic response of the material, while $a_{f}$ and $b_{f}$ characterize the additional stiffness in the fiber direction. $I_{1}$ denotes the first deviatoric strain invariant, representing the trace of the modified right Cauchy-Green deformation tensor. $I_{4f}$ is a pseudo-invariant defined as $A_{f}\cdot C\cdot A_{f}$ , where $A_{f}$ is the fiber direction vector in the reference configuration. For clarity, all remaining symbols and parameters are summarized in the Nomenclature section.

To model the active contraction behavior of myocardial tissue, the LHM adopted a time-varying elastance-based active material model, as given in Equation 6,

$$\sigma_{af}\left(t,E_{ff}\right)=\frac{T_{\max}}{2}\frac{{Ca}_{0}^{2}}{{Ca}_{0}^{2}+E{Ca}_{50}^{2}\left(E_{ff}\right)}\left(1-\cos\left(\omega \left(t,E_{ff}\right)\right)\right),$$ (6)

where $E_{ff}$ is the fiber-direction Green–Lagrange strain component and $T_{\max}$ represents the maximum allowable isometric active tension which occurs when the SL is at its maximum and the intracellular ${Ca}^{2+}$ concentration reaches its peak, ${{Ca}_{0}}_{\max}$ . The ${Ca}^{2+}$ sensitivity that depends on length and the internal variable is defined by:

$$E{Ca}_{50}\left(E_{ff}\right)=\frac{{{Ca}_{0}}_{\max}}{\sqrt{e^{B\left(L\left(E_{ff}\right)-L_{0}\right)}-1}},$$ (7)

where B was a constant, $L_{0}$ represented the SL at which no active tension develops. In this formulation, both $L_{0}$ and $L_{r}$ are inherently embedded within the length-dependent ${Ca}^{2+}$ sensitivity function. The current sarcomere length $L\left(E_{ff}\right)$ , which governs the SL–dependent shift in ${Ca}^{2+}$ responsiveness, is derived from the fiber stretch applied to the initial reference length $L_{r}$ . Meanwhile, $L_{0}$ serves as a threshold below which no active tension develops. As $L\left(E_{ff}\right)$ exceeds $L_{0}$ , the required ${Ca}^{2+}$ concentration $E{Ca}_{50}$ to reach half-maximal tension decreases, reflecting the increased ${Ca}^{2+}$ sensitivity at longer sarcomere lengths. This length-dependent modulation of ${Ca}^{2+}$ sensitivity is central to replicating the Frank-Starling mechanism and forms a key component in the myocardial activation model.

Furthermore, the temporal evolution of activation is represented as a function of time and fiber strain, as given in Equation 8.

$$\omega \left(t,E_{ff}\right)=\left\{\begin{array}{l} \pi \frac{t}{t_{0}}\;when\;0\leq t<t_{0}\\ \pi \frac{t-t_{0}+t_{r}\left(L\left(E_{ff}\right)\right)}{t_{r}}\;when\;t_{0}\leq t<t_{0}+t_{r}\left(L\left(E_{ff}\right)\right)\\ 0\;when\;t\geq t_{0}+t_{r}\left(L\left(E_{ff}\right)\right) \end{array}\right..$$ (8)

In Equation 3, the duration of relaxation, $t_{r}$ denoted a linear function of SL:

$$t_{r}\left(L\right)=mL+b,$$ (9)

where m and b are constants. SL is in general, a function of 3D position in the LV wall that is related to fiber strain by the following relation:

$$L\left(E_{ff}\right)=L_{r}\sqrt{2E_{ff}+1},$$ (10)

where $L_{r}$ is initial SL in the unloaded reference configuration. Based on the experimental results of Rodriguez et al., sarcomere length was assumed to vary linearly from 1.78 μm at the endocardium to 1.91 μm at the epicardium (Rodriguez et al., 1993). Therefore, the $L_{r}$ value for the LV in the all models was set to 1.85 µm (ter Keurs et al., 2008). Walker et al. also employed these values from rodent experiments in their finite element analysis of myocardial infarction in ovine hearts (Walker et al., 2005). This setting appears to be based on the assumption that sarcomere characteristics are largely conserved across mammalian species (Mojumder et al., 2023). In this study, we varied only the sarcomere-length–related parameters $L_{r}$ and $L_{0}$ . All other active-stress parameters were adopted directly from the default parameter set implemented in the Living Heart Model (time-varying elastance formulation), and no additional parameter fitting was performed in the present work. These default values are consistent with those reported in previous Living Heart model studies (Walker et al., 2005; Mojumder et al., 2023; Goetz et al., 2024). The parameter values used in our simulations were $T_{\max}=0.1$ , ${Ca}_{0}=4.35$ , ${{Ca}_{0}}_{\max}=4.35$ , B=4750 , $t_{0}=0.1$ , m=1048.9 , and b=−1.429 (Walker et al., 2005; Mojumder et al., 2023; Goetz et al., 2024).

### 2.3 Simulation scenario

**FIGURE 1:**
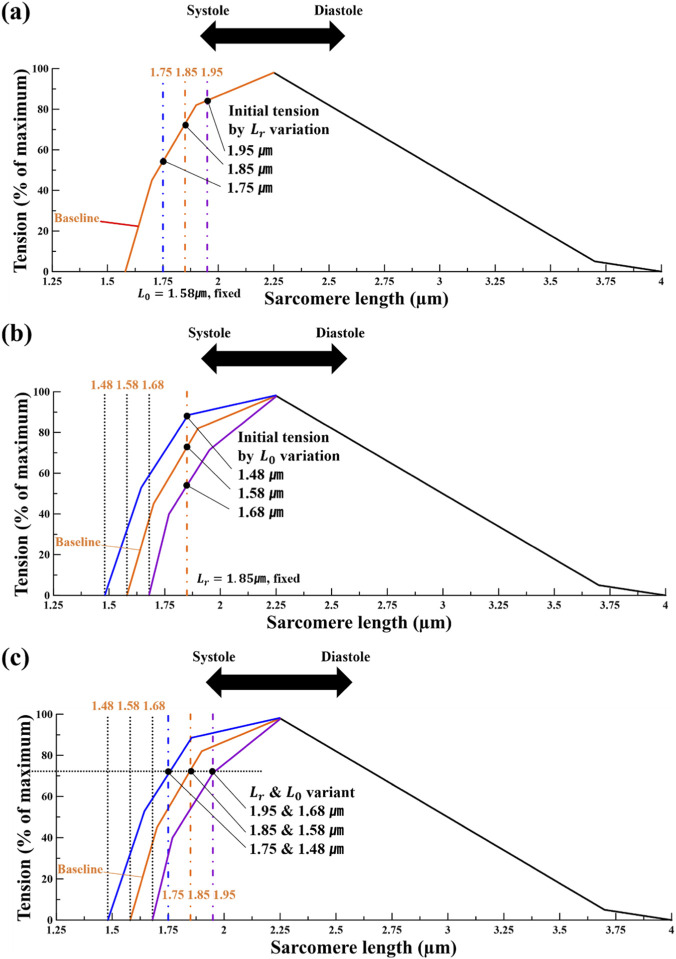
Conceptual schematic of the model-based sarcomere length -tension relationship used to motivate the simulation scenarios. An increase in L_r_ elevates the initial tension level **(a)**, whereas changes in L_0_ alter the overall curve shape **(b)**. Simultaneous variation of both parameters is shown in **(c)**, adapted with modifications from Shiels and White (2008).

To investigate the role of sarcomere length parameters in myocardial mechanics, we designed three main types of simulation scenarios: (1) varying $L_{r}$ , (2) varying $L_{0}$ , and (3) simultaneously varying both $L_{r}$ and $L_{0}$ while maintaining a fixed difference between them, referred to as sarcomere length difference (SLD, $L_{r}-L_{0}$ ). Each scenario was designed to isolate the mechanical effects of SL changes and assess how they influence active tension generation and global cardiac performance. In this context, $L_{r}$ is involved in both $E{Ca}_{50}$ modulation and contraction duration ( $t_{r}$ ) within the TVE model formulation as described in Section 2.2, whereas $L_{0}$ modulates only $E{Ca}_{50}$ , which defines the steepness and midpoint of the calcium–tension curve. Based on the length–tension formulation, varying $L_{r}$ shifts the sarcomere’s operating range along a given length–tension curve without changing its shape, whereas varying $L_{0}$ translates the curve by shifting the tension-free reference length. These distinct roles motivated the selection of parameter ranges to probe potentially different mechanical responses; Figure 1 provides a schematic illustration of the expected shifts in the length–tension relationship under each scenario. The default value for $L_{r}$ in the Living Heart Model is 1.85 µm, and the default value for $L_{0}$ is 1.58 μm. These defaults follow the baseline parameter values used by Walker et al. in their active stress law, and the same values are adopted as the default settings in the Living Heart Model (Walker et al., 2005). Using these default values as a reference, we designed three simulation scenarios, summarized in Table 1 and described below.

**TABLE 1:** Living heart model simulation scenarios with modified SL variables $L_{r}$ and $L_{0}$ .

S0 series	S01	S02	S00^a^	S03	S04
$L_{r}$ (㎛), variant	1.75	1.8	1.85	1.9	1.95
$L_{0}$ (㎛), fixed	1.58	1.58	1.58	1.58	1.58
$L_{r}-L_{0}$ (㎛), variant	0.17	0.22	0.27	0.32	0.37

S1 series	S11	S12	S00	S13	S14
$L_{r}$ (㎛), fixed	1.85	1.85	1.85	1.85	1.85
$L_{0}$ (㎛), variant	1.48	1.53	1.58	1.63	1.68
$L_{r}-L_{0}$ (㎛), variant	0.37	0.32	0.27	0.22	0.17

S2 series	S21	S22	S00	S33	S34
$L_{r}$ (㎛), variant	1.75	1.8	1.85	1.9	1.95
$L_{0}$ (㎛), variant	1.48	1.53	1.58	1.63	1.68
$L_{r}-L_{0}$ (㎛), fixed	0.27	0.27	0.27	0.27	0.27

^a^ S00 denotes the control parameters, which are taken as the reference for all comparisons.

#### 2.3.1 Scenario 1 – S0

In Scenario 1, the $L_{0}$ was fixed while $L_{r}$ was varied, allowing for comparative validation with previous research results. The scenario was named S0, indicating that it maintains the original research framework. $L_{0}$ was set to 1.58 μm, as established by Guccione and in the LHHM based on experimental results by ter Keurs et al., and $L_{r}$ was varied across the values 1.75, 1.8, 1.85, 1.9, and 1.95 μm (ter Keurs et al., 1980; Guccione and McCulloch, 1993). The range of $L_{r}$ values fell within the scope of previous studies, allowing for a comparison to validate the reliability of this study’s results (ter Keurs et al., 1980; Guccione and McCulloch, 1993). The simulations were performed by manipulating the SL in the LV of the LHM using the LHHM materials model. The differences between $L_{r}$ and $L_{0}$ , i.e., SLDs were 0.17, 0.22, 0.27, 0.32, and 0.37 μm, respectively. Figure 1a schematically depicts Scenario 1: with $L_{0}$ , fixed at 1.58 µm, varying $L_{r}$ from 1.75 to 1.95 µm shifts the operating point along the same length–tension curve. Accordingly, Scenario 1 was designed to represent a shift of the sarcomere operating range along a single length–tension curve as $L_{r}$ changes.

#### 2.3.2 Scenario 2 – S1

In this scenario, $L_{r}$ was fixed and $L_{0}$ was varied. Previous studies observed that the experimental values generally ranged between 1.5 and 1.6 μm, with the minimum length of the sarcomere reported to be at least 1.4 μm (van Heuningen et al., 1982; Krueger and Tsujioka, 1986; ter Keurs et al., 1980). Therefore, the range was set to 1.48, 1.53, 1.58, 1.63, and 1.68 μm. $L_{r}$ was fixed at 1.85 μm. The SLDs were set at 0.17, 0.22, 0.27, 0.32, and 0.37 μm, listed from the smallest to the largest, which is the same as in Scenario 1 (S0). This provides a common basis for comparing S0, S1, and S2. Figure 1b provides a schematic representation of Scenario 2: with $L_{r}$ fixed at 1.85 µm, varying $L_{0}$ translates the length–tension curve leftward or rightward relative to the baseline ( $L_{0}=1.58\;µm$ ) thereby altering the tension-free reference length. Conversely, increasing $L_{0}$ to 1.68 µm shifted the curve rightward (green), indicating a lower initial tension. This scenario was constructed in accordance with reported physiological ranges and prior length–tension observations in muscle, treating $L_{0}$ as a parameter that governs the tension-free reference length and thus the baseline activation threshold within the model (Shiels and White, 2008).

#### 2.3.3 Scenario 3 – S2

The difference between the original values of $L_{r}$ and $L_{0}$ , i.e., SLD was 0.27 μm. Therefore, by fixing the difference between the two SLs at 0.27 μm, five sets were created by altering both the lengths. The SLD values, listed in order, are 1.95–1.68, 1.9–1.63, 1.85–1.58, 1.8–1.53, and 1.75–1.48 μm. In contrast to Scenarios 1 and 2, which vary $L_{r}$ or $L_{0}$ individually, this setup varies both parameters simultaneously while holding SLD fixed, thereby decoupling absolute-length shifts from changes ${Ca}^{2+}$ sensitivity at contraction onset. According to Equation 7, $E{Ca}_{50}$ is defined as a function of $L\left(E_{ff}\right)-L_{0}$ . At the onset of contraction, i.e., when $E_{ff}$ = 0, $L\left(E_{ff}\right)$ corresponds to $L_{r}$ , making the term $L\left(E_{ff}\right)-L_{0}$ equal to $L_{r}-L_{0}$ . By fixing SLD across all sets, the model’s baseline ${Ca}^{2+}$ sensitivity at contraction onset is held constant, enabling isolation of the effects of absolute sarcomere length shifts. As shown in Figure 1c, each pair of $L_{r}$ and $L_{0}$ values is represented by the same color set; for example, the blue curve corresponds to $L_{r}$ = 1.75 µm and $L_{0}$ = 1.48 µm, the red curve corresponds to $L_{r}$ = 1.85 µm and $L_{0}$ = 1.58 µm, and the green curves (solid and dashed) correspond to $L_{r}$ = 1.95 µm and $L_{0}$ = 1.68 µm. Under the constant-SLD design, all parameter sets share the same initial value of SLD at contraction onset, which corresponds to an identical starting point on the schematic length–tension curve in Figure 1c.

### 2.4 Data analysis

Based on the simulation scenarios described above, we performed two types of analyses. First, we conducted 1 × 1 × 1 unit-cube verification tests to confirm that the passive constitutive response and active stress development are correctly reproduced in Abaqus/Explicit under the Living Heart Model settings. Second, we performed organ-level left-heart simulations to quantify global hemodynamic outputs and local myocardial mechanics.

#### 2.4.1 Unit-cube verification for the passive constitutive response under biaxial deformation

To verify the passive constitutive response, we analyzed a unit cube with edge lengths of 1 using a single C3D8R element, an 8-node linear hexahedral continuum element with reduced integration in Abaqus/Explicit. The simulated time interval of the explicit step was 0.5 s, and automatic time incrementation was used. The cube geometry and local material directions were defined so that the global axes coincide with the fiber, sheet, and sheet-normal directions. The electrical potential was initialized to the resting value of −80 mV and kept constant throughout this passive verification so that no active stress developed. Biaxial deformation was imposed by prescribing displacements on the faces normal to the fiber and sheet directions. Displacements were applied to achieve the target fiber stretch ratio and sheet stretch ratio, up to a maximum stretch ratio of 1.3. The opposite faces were constrained to remove rigid-body motion. Faces normal to the sheet-normal direction were traction-free, allowing the sheet-normal stretch to develop naturally.

Under this homogeneous biaxial deformation setting, analytical expressions for the Cauchy stress components in the fiber and sheet directions can be written as follows, as obtained by specializing the Holzapfel–Ogden formulation (Holzapfel and Ogden, 2009) to a homogeneous biaxial stretch under incompressibility as follow,

$$\sigma_{ff}=a\exp\left[b\left(I_{1}-3\right)\right]\left(\lambda_{f}^{2}-\lambda_{n}^{2}\right)+2a_{f}\left(I_{4f}-1\right)\exp\left[b_{f}{\left(I_{4f}-1\right)}^{2}\right]\lambda_{f}^{2},$$ (11)

$$\sigma_{ss}=a\exp\left[b\left(I_{1}-3\right)\right]\left(\lambda_{s}^{2}-\lambda_{n}^{2}\right)+2a_{s}\left(I_{4s}-1\right)\exp\left[b_{s}{\left(I_{4s}-1\right)}^{2}\right]\lambda_{s}^{2}.$$ (12)

For the unit-cube verification, the passive parameter values were set to those provided in the LHM verification files: a=0.004 , b=8.0 , $a_{f}=0.005$ , $b_{f}=5.0$ , $a_{s}=0.002$ , and $b_{s}=2.0$ . $\lambda_{f}$ , $\lambda_{s}$ and $\lambda_{n}$ denote the principal stretches in the fiber, sheet, and sheet-normal directions, respectively. If the material is nearly incompressible, then given a prescribed stretch in the fiber and sheet directions, $\lambda_{n}$ can be calculated as follows,

$$\lambda_{n}=\frac{1}{\lambda_{f}\lambda_{s}}.$$ (13)

**FIGURE 2:**
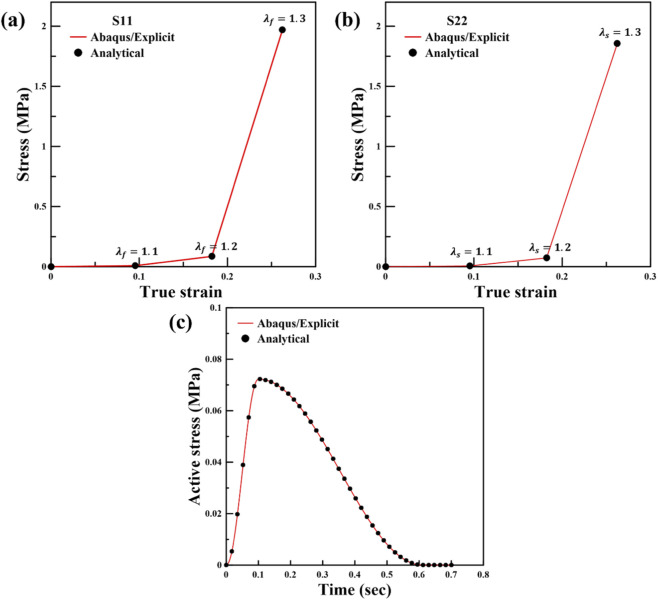
Verification results: analytical solutions are compared with Abaqus/Explicit predictions for passive $\sigma_{ff}$ **(a)**, passive $\sigma_{ss}$ **(b)**, and time-varying active stress **(c)**.

The Abaqus/Explicit outputs for $\sigma_{ff}$ and $\sigma_{ss}$ were compared against the analytical values from Equations 11–13 for prescribed stretch ratios of 1.10, 1.20, and 1.30. Figure 2 plots the corresponding stress components, $S_{11}$ (fiber direction) and $S_{22}$ (sheet direction), obtained from the analytical and numerical evaluations, confirming close agreement over the tested stretch range.

#### 2.4.2 Unit-cube verification of the active myocardial material response

To verify active stress development, we performed the standard Living Heart Model active-material unit-cube benchmark using a single C3D8R element with dimensions of 1 × 1 × 1 in Abaqus/Explicit. The total analysis time was 0.7 s with automatic time incrementation. The electrical potential was initialized to −79 mV, and the activation threshold was also set to −79 mV so that active stress development begins immediately at the start of the simulation. Mechanical boundary conditions were applied to hold the cube kinematics fixed in order to isolate the active stress response. All nodes were constrained in the fiber direction. Additional constraints were applied in the sheet and sheet-normal directions to remove rigid-body motion. The time-varying fiber active stress response obtained from Abaqus/Explicit was compared against the analytical or reference response provided in the Living Heart Model verification procedure. The comparison is reported in Figure 2c and confirms correct active-stress behavior under the settings used in this study.

#### 2.4.3 Organ-level left-heart simulations and extracted outputs

For the LHM-based simulations, the left heart underwent a repeated cycle consisting of a 0.3-s preload phase followed by a 0.5-s beat phase and a 0.5-s recovery phase. The two-chamber left-heart finite-element model comprised 145,818 nodes and 93,811 elements, and the spatial discretization followed the default LHM mesh and element formulations. Time integration was performed in Abaqus/Explicit with an automatically determined stable time increment, and field outputs were written at regular frame intervals using identical settings across all cases. The simulation results provided the pressure–volume relation of the LV, including the end-systolic volume (ESV) and end-diastolic volume (EDV), from which SV, LVEF, and CO were calculated. Additionally, the strain evolution resulting from various regions of the LV was used to obtain average peak strain data (Hayabuchi et al., 2023; Singh et al., 2021). Furthermore, time-varying fiber active stress and fiber stretch were extracted at element integration points to characterize local myocardial contractile behavior in greater detail. These outputs were also used to quantify deviations from literature-reported normal ranges and to identify the most abnormal scenarios in terms of cardiac function and APS.

## 3 Results

### 3.1 Verification and analysis of the myocardial material model

In this section, the myocardial material models were verified by comparing the numerical Abaqus/Explicit outputs with analytical solutions. Specifically, Figures 2a,b compared the analytical and numerical Cauchy stress components predicted by the passive material model under prescribed stretch states. In addition, Figure 2c compared the time-varying active stress response obtained from the active model with the corresponding analytical TVE predictions. The close agreement in these comparisons confirms that the passive and active material implementations are computed accurately. Building on this verification, the remainder of this section provides a detailed analysis of how the temporal active-stress behavior at the myocardial material level varies with changes in $L_{r}$ and $L_{0}$ , with a particular emphasis on the active stress response, which is the primary focus of this study.

**FIGURE 3:**
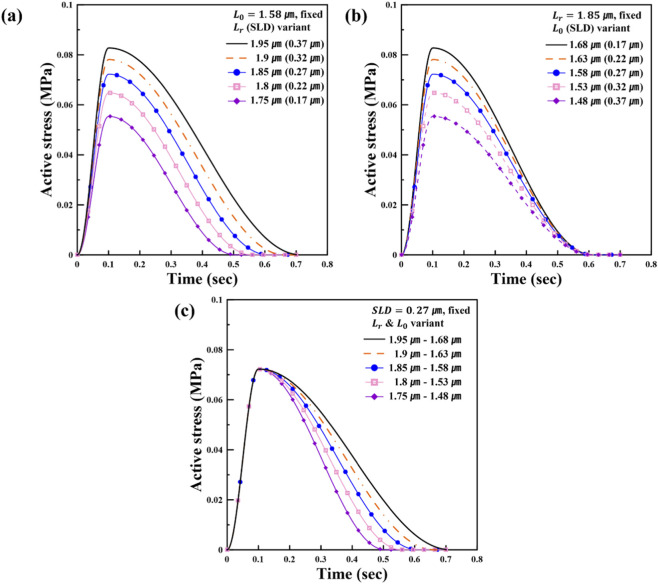
Effect of SL variation on the time course of isometric active stress development in the fiber direction: **(a)** changes in $L_{r}$ , **(b)** changes in $L_{0}$ , and **(c)** simultaneous changes in both $L_{r}$ and $L_{0}.$

From the length–tension curves in Figure 1, the changes in active tension with respect to variations in $L_{r}$ and $L_{0}$ were identified. In Figure 1a, with $L_{0}$ fixed, an increase in $L_{r}$ led to higher initial tension. In Figure 1b, with $L_{r}$ held constant, lowering $L_{0}$ increased the initial tension. From the perspective of SLD, an increase in SLD was commonly associated with an increase in active tension; however, the source of this increase differed. Varying $L_{r}$ ​ shifts the starting point along the same length–tension curve, whereas varying $L_{0}$ changes the curve itself, resulting in different initial active tensions. In Figure 1c, when $L_{r}$ and $L_{0}$ were varied simultaneously so that the SLD remained the same, the initial tension at the starting $L_{r}$ was nearly identical across cases. These findings are consistent with prior reports describing length–tension relationship in cardiac sarcomeres and align with the predicted shifts in the curves based on changes in $L_{r}$ and $L_{0}$ . Supporting evidence for these observations is provided in Figure 3 (Shiels and White, 2008).

At the myocardial material level, we first examined how the time-varying active stress response changes with the reference sarcomere length parameter $L_{r}$ using the verification procedure described above, and the resulting active stress waveform is shown in Figure 3a. The results are consistent to those from prior research and align well with the analytical results of the TVE model. As $L_{r}$ increased, the peak active stress rose and the duration of contraction (DOC) extended, in agreement with the findings of Guccione and McCulloch (1993), Guccione et al. (1993). This agreement strengthens confidence that the Abaqus/Explicit implementation captures the expected changes in peak active stress and duration of contraction with varying $L_{r}$ , providing a reliable basis for the subsequent analyses. Based on this premise, the results in Figures 3b,c were analyzed. An increase in $L_{r}$ , with $L_{0}$ held constant, leads to a larger SLD, which enhances both peak active stress and DOC, as shown in Figure 3a. This behavior is consistent with the Frank-Starling mechanism, whereby SL–dependent (stretch-associated) ${Ca}^{2+}$ sensitivity enhances contractile force. In contrast, Figure 3b illustrates the effect of varying $L_{0}$ while maintaining a fixed $L_{r}$ at 1.85 μm. As $L_{0}$ decreased, SLD increased, which led to an increase in peak active stress. However, unlike $L_{r}$ variation shown in Figure 3a, DOC remained unchanged. This distinction is clearly observable in the transition from the blue to the black curve in Figure 3b, where a reduction in $L_{0}$ increases SLD and elevates peak stress without affecting the temporal duration of contraction. This contrast emphasizes the distinct physiological roles of $L_{r}$ and $L_{0}$ in the active stress generation model. Specifically, $L_{0}$ appears only in the ${Ca}^{2+}$ sensitivity threshold (Equation 7), modulating the ease of activation (*via* $E{Ca}_{50}$ ) without affecting the relaxation time. Meanwhile, $L_{r}$ is embedded in both the fiber stretch-dependent sarcomere length (Equation 10) and the length-dependent relaxation time (Equation 9), thereby influencing both the magnitude and the temporal duration of active stress. These findings are further reinforced in Figure 3c, where both $L_{r}$ and $L_{0}$ were varied simultaneously while keeping SLD fixed at 0.27 μm. Despite different absolute values of $L_{r}$ and $L_{0}$ , the peak active stress remained constant due to the same SLD, confirming that SLD alone governs peak force generation. While the peak active stress was mainly determined by SLD, DOC continued to increase with $L_{r}$ , likely due to its effect on the relaxation function. This suggests that $L_{r}$ contributes not only to contractile magnitude *via* SLD but also to the temporal pattern of contraction. Taken together, and in light of the TVE formulation, these results validate that $L_{0}$ serves as a ${Ca}^{2+}$ sensitivity threshold, whereas $L_{r}$ regulates the dynamic response of myocardial contraction. Furthermore, this finding aligns with the predictive curves in Figure 1, indicating that an increase in SLD elevates the initial active tension, thereby leading to greater active stress.

### 3.2 Myocardial mechanical response in the LHM

Three-dimensional LV strain during systole was analyzed to characterize myocardial deformation. Radial, circumferential, and longitudinal strains were computed from the ABAQUS/Explicit solution of the Living Heart Model using nodal coordinates, and regional variations were assessed across the basal, equatorial, and apical levels as well as among anterior, posterior, septal, and lateral segments. Strains were reported as directional nominal (engineering) strains, defined as the fractional change in a direction-specific length measure between ED and ES, as given in Equation 14,

$$e=\frac{l_{ES}-l_{ED}}{l_{ED}}=\lambda -1,$$ (14)

where l denotes radial wall thickness, circumferential length, or longitudinal length obtained from predefined node sets, and e is used to distinguish the present nominal strain from the Green–Lagrange strain components E reported in the reference study (Moore et al., 2000). Strain was evaluated from ED to ES in the fifth cardiac cycle. The results were compared with the systolic ranges reported by Moore et al. (2000) from MR tissue tagging in healthy volunteers. Because Moore et al. reported Green–Lagrange strain components E , their values were converted to the nominal strain measure used in this study using $e=\sqrt{2E+1}-1$ prior to comparison. The effects of variations in $L_{r}$ and $L_{0}$ on strain evolution were evaluated, and the strain analysis was interpreted together with active stress and fiber stretch distributions.

#### 3.2.1 Scenario S00 – Baseline

**FIGURE 4:**
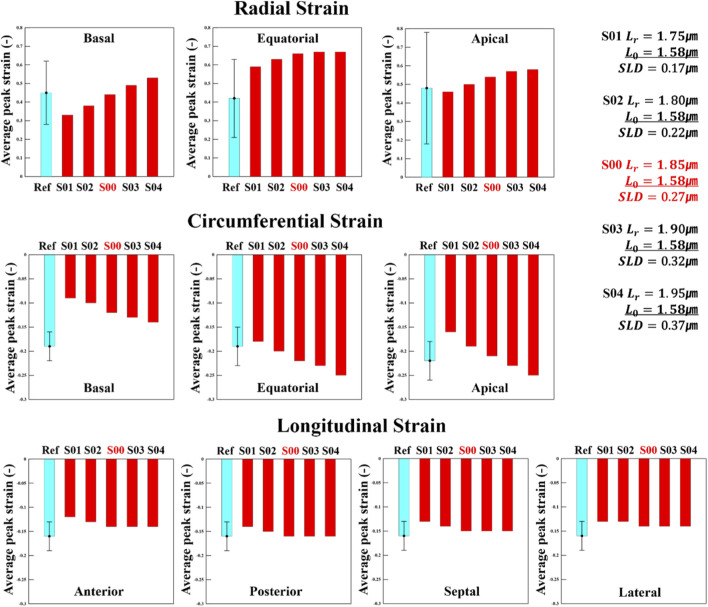
Changes in radial strain, circumferential strain, and longitudinal strain for the S0 scenario series (fifth cardiac cycle): The S0 series represents scenarios in which $L_{r}$ was varied relative to the control case, S00. The light-blue reference bars represent literature-reported normal values of average peak strain (APS; mean ± SD), and the error bars indicate ±SD of these experimental statistics. All S0 scenarios (including S00) are simulation outputs shown as single values.

**FIGURE 5:**
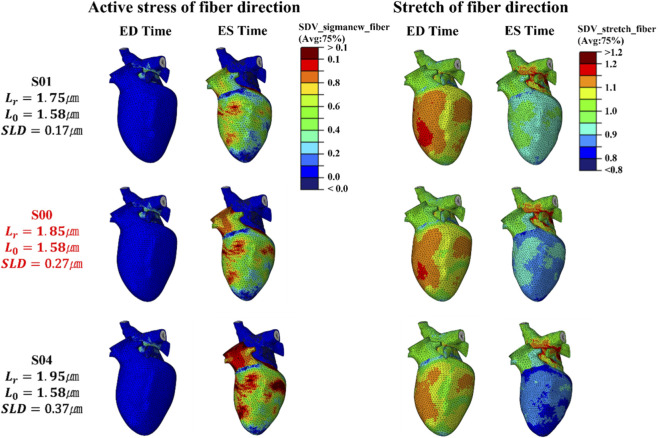
Magnitude of active stress and stretch in the fiber direction at the integration point for the S0 series, evaluated at ED and ES (fifth cycle, stress unit: MPa).

**FIGURE 6:**
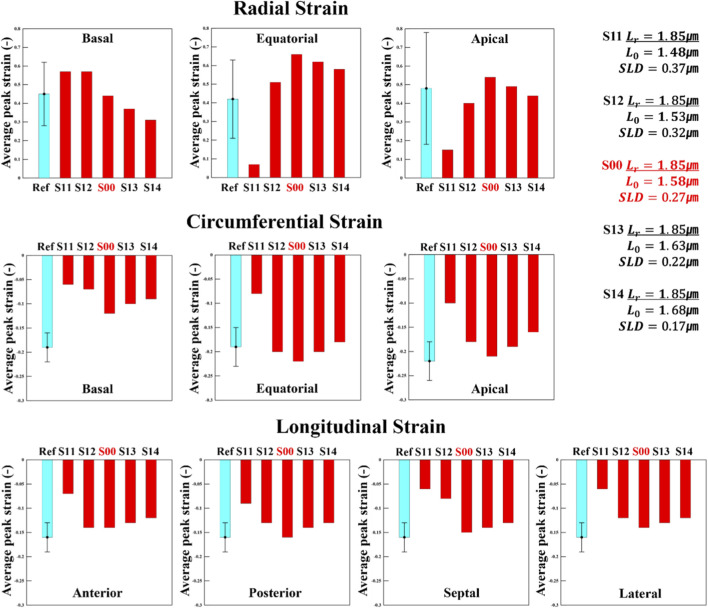
Changes in radial strain, circumferential strain, and longitudinal strain for the S1 scenario series (fifth cardiac cycle). The S1 series represents scenarios in which $L_{0}$ was varied relative to the control case, S00. The light-blue reference bars represent literature-reported normal values (mean ± SD), and the error bars indicate ±SD of these experimental statistics.

**FIGURE 7:**
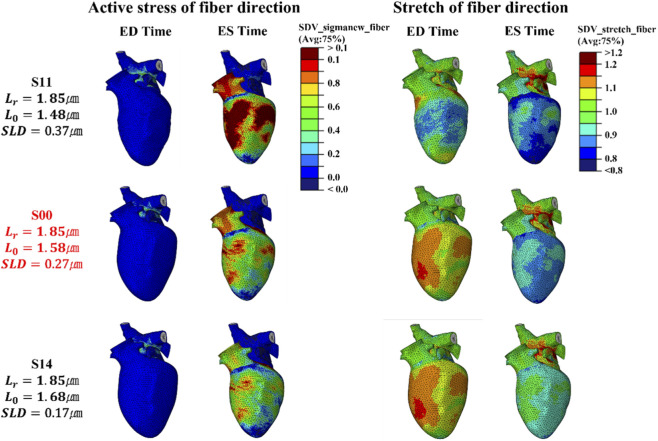
Magnitude of active stress and stretch in the fiber direction at the integration point for the S1 series, evaluated at ED and ES in the fifth cycle (stress unit: MPa), compared with the control case, S00.

**FIGURE 8:**
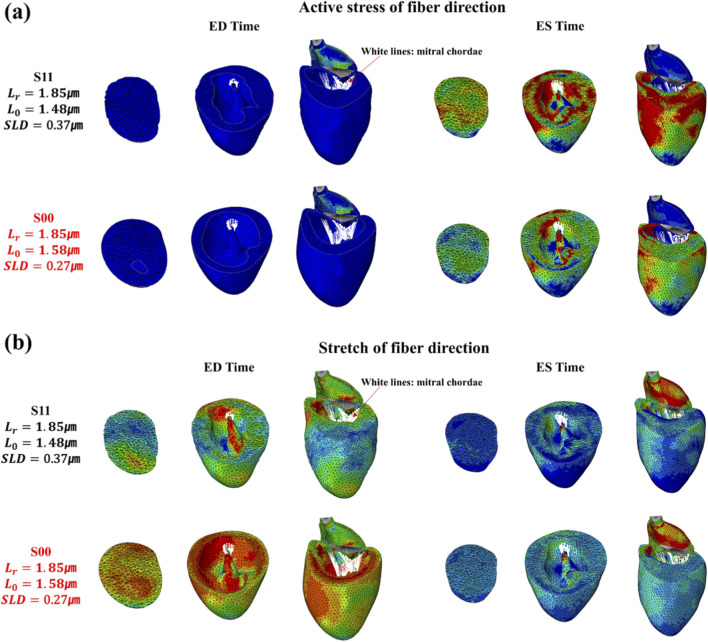
Comparison of **(a)** fiber-direction active stress and **(b)** fiber-direction stretch at ED and ES (fifth cycle, stress unit: MPa) between S11 and S00, illustrated for basal, equatorial, and apical regions.

**FIGURE 9:**
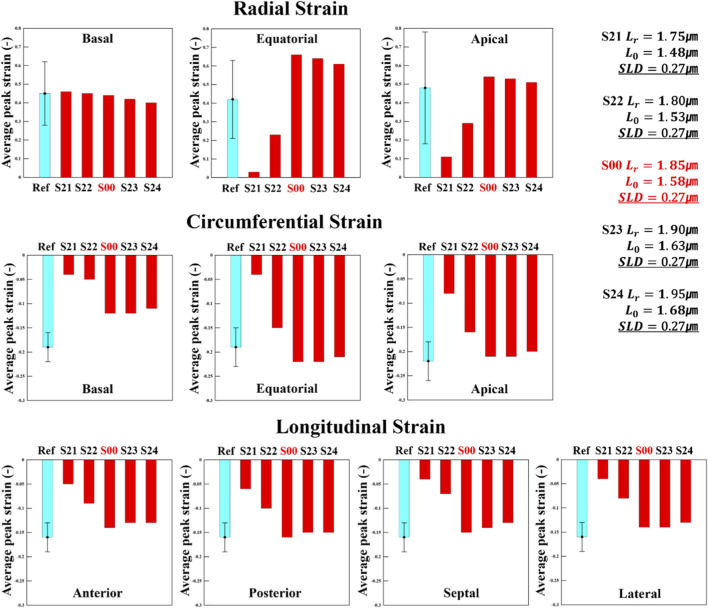
Changes in radial strain, circumferential strain, and longitudinal strain for the S2 scenario series (fifth cardiac cycle). The S2 series represents scenarios in which $L_{r}$ and $L_{0}$ were varied simultaneously relative to the control case, S00 while keeping the sarcomere-length difference (SLD) constant. The light-blue reference bars represent literature-reported normal values (mean ± SD), and the error bars indicate ±SD of these experimental statistics.

**FIGURE 10:**
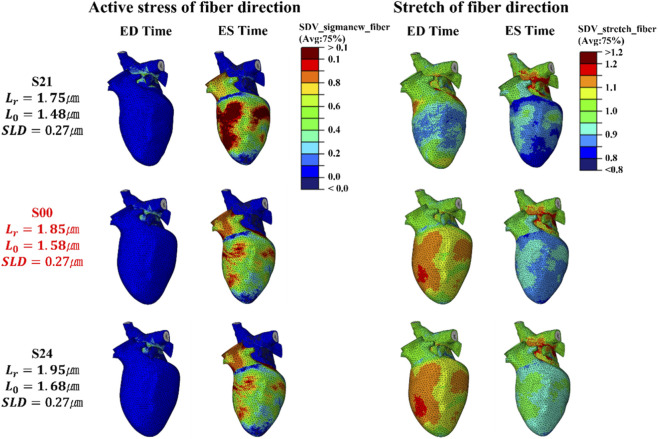
Magnitude of active stress and stretch in the fiber direction at the integration point for the S2 series, evaluated at ED and ES in the fifth cycle (stress unit: MPa), compared with the control case, S00.

The baseline values of $L_{r}$ and $L_{0}$ are 1.85 μm and 1.58 μm, respectively, yielding an SLD of 0.27 μm. As noted earlier, this configuration represents a normal male heart in the LHM, constructed by applying the findings of Rodriguez et al., Guccione et al., and ter Keurs et al., and therefore serves as the control scenario in this study (ter Keurs et al., 1980; Rodriguez et al., 1993; Guccione and McCulloch, 1993). Examining the strain change from ED time to ES time in S00—used as the control—reveals that it appears in Figures 4, 6, 9, and that most values fall within, or only slightly outside, the healthy reference range. This reference range is indicated by the cyan bar on the left side of each graph (Moore et al., 2000). This indicates that the model reproduces the characteristics of a healthy human heart well. A more detailed discussion will be provided in the next paragraph when comparing this with other scenarios. Meanwhile, Figures 5, 7, 8, 10 focus on the left heart, particularly the left ventricle, showing the distributions of both active stress and stretch of the fiber direction from ED time to ES time.

#### 3.2.2 Scenario S0 – Variable $L_{r}$ , fixed $L_{0}$

Figure 4 shows the analysis of the radial strain of the S0 series by dividing the LV into three regions: the basal region, which reflects deformation near the atrium at the upper part of the heart; the equatorial region, associated with deformation in the middle section of the LV; and the apical region, which focuses on deformation at the apex of the heart. This figure illustrates the APS for each of these regions (Loncaric et al., 2018). Radial strain indicates the change in thickness of the heart wall, where higher positive values imply greater contraction and thickening (Jeung et al., 2012). In this scenario, changes in $L_{r}$ exhibited a clear trend without appearing abnormal. $L_{r}$ increased from 1.75 μm in S01 to 1.95 μm in S04, showing a consistent trend of increase in radial strain across all three regions—basal, equatorial, and apical—indicating that radial strain is proportional to $L_{r}$ . Figure 4 also illustrates the strain evolutions in the circumferential direction, reflecting the deformations as the heart contracts and narrows circumferentially (Jeung et al., 2012). A larger negative value indicates greater deformation, suggesting significant contraction of the heart in this direction. For circumferential strain, the S0 scenario also showed a distinct trend: as $L_{r}$ increased, strain became more negative in all three regions. This indicates a much greater circumferential shortening ratio, which is consistent with the results in Figures 1, 3 showing that larger $L_{r}$ ​ values generate greater contractile force. The APS– $L_{r}$ relationships were quantified by linear regression and are summarized in Supplementary Table S1, confirming monotonic increases in radial APS and increasingly negative circumferential APS with increasing $L_{r}$ . Supplementary Table S1 further shows that circumferential APS exhibited the highest $R^{2}$ , indicating the strongest linear dependence on $L_{r}$ while radial APS also showed high $R^{2}$ values, although slightly lower than those of the circumferential component.

Finally, to discuss the strain evolution in the longitudinal direction (Amzulescu et al., 2019), Figure 4 presents the APS in this direction for four regions. The results of APS for the septal, anterior, lateral, and posterior regions in S0 are shown respectively. The septal and anterior regions are among the areas where myocardial infarction frequently occurs; hence, detecting abnormal deformation patterns in these regions can indicate myocardial damage. Moreover, observing lateral strain evolution can provide important information about the overall pumping function of the LV, and monitoring changes in posterior longitudinal strain is also a non-invasive method to predict myocardial damage. Therefore, analyzing these factors is a critical aspect of cardiac research. For S0, longitudinal APS became more negative with increasing $L_{r}$ in the septal, lateral, and posterior regions, indicating greater longitudinal shortening. This relationship was comparatively weaker than that observed for circumferential strain, as reflected by lower $R^{2}$ values in Supplementary Table S1, supporting our description of a slight trend in the longitudinal direction.

When these region-specific strain results are analyzed together with the distributions of active stress and fiber-direction stretch shown in Figure 5, a clear pattern emerges. Increasing $L_{r}$ produces larger stress distributions at ES, with particularly high stresses observed in the basal region and near the aortic junction. This trend is most clearly visible in scenario S04, where the red contour areas highlight the regions of elevated stress. The stretch contours are represented by a color scale in which blue corresponds to myocardial shortening and red corresponds to elongation. In scenario S01, the contours at ED appear more prominently in red, indicating greater elongation and showing that the myocardium is more relaxed at the end of diastolic filling. This behavior can be explained by the length–tension relationship: when is lower, the sarcomere operates further to the left on the curve, a region associated with weaker force generation, as illustrated in Figure 1a. As a result, the myocardium at ED is in a relatively slack state, allowing more elongation during filling and thus appearing more relaxed. However, despite this larger ED stretch, the subsequent contraction from ED to ES was less pronounced because the sarcomere failed to reach the optimal length for maximal force generation. Consequently, contractile force was weaker, leading to smaller net shortening. This reduced contractility is reflected in the PV loop, where the difference between EDV and ESV was smaller than in the control scenario, confirming the reduction in SV observed for S01. In contrast, S04, with a higher initial $L_{r}$ ​, starts with higher initial tension and is therefore less relaxed during filling, resulting in a reduced EDV. However, the much stronger contractile force produces a substantially greater reduction in ESV, leading to an increased SV. This change in cardiac function is further confirmed in Section 3.3.

#### 3.2.3 Scenario S1 – Fixed $L_{r}$ , variable $L_{0}$

Figure 6 shows the regional radial strain for the S1 series. In this case, because $L_{0}$ was varied, the SL length–tension curve was fundamentally altered as illustrated in Figure 1b, leading to a much more complex set of results. When $L_{0}$ increased from the control scenario S00 toward S13 and S14, the results appeared close to normal, and the strain values consistently decreased compared with S00. This monotonic decrease with increasing $L_{0}$ in the high segment is quantified by piecewise linear regression of APS *versus* $L_{0}$ and is summarized in Supplementary Table S1, where the high-segment fits show $R^{2}$ values close to unity in most regions. This trend can be explained by the fact that, with $L_{r}$ fixed, a larger $L_{0}$ reduces the difference between $L_{r}$ and $L_{0}$ , that is, the SLD. This relationship is essentially the same as what was observed between S01 or S02 and S00 in the S0 series, and the strain tendencies were found to be correspondingly similar. A comparable pattern can also be seen in Figures 3a,b, where the magnitude of active stress is determined primarily by the SLD.

However, excluding the basal region, reducing $L_{0}$ to 1.48 μm caused a pronounced reduction in strain. Compared with S00, lowering $L_{0}$ decreased strain, and increasing $L_{0}$ also decreased strain. This pattern was similarly observed in the circumferential and longitudinal directions (Figure 6). This non-monotonic response around S00 is also captured by the piecewise regression in Supplementary Table S1. Specifically, the below-baseline segment S11–S12–S00 and the above-baseline segment S00–S13–S14 yield opposite slope directions in several regions, indicating that strain decreases when $L_{0}$ deviates from 1.58 μm toward either lower or higher values. The mechanisms behind the strain changes with decreased and increased $L_{0}$ appear to differ, and we analyzed these using Figures 1, 7. As shown in Figure 1B, lowering $L_{0}$ shifts the length–tension curve to the left. In this state, not only the initial $L_{r}$ but also all subsequent stretches occur at higher tension, resulting in the myocardium reaching a relatively high activation level early in the beat cycle. Consequently, in Figure 7, S11 shows almost no relaxation at ED, indicating diastolic dysfunction; the equatorial region is even in a contracted state. Since the myocardium is not relaxed initially, further shortening produces little strain change. Nevertheless, because active tension is high from the outset, the stress distribution is greatly elevated, suggesting that a myocardium that has not been stretched is being strongly compressed. Although further evidence can be found in the pressure–volume (PV) loop, it was observed that with repeated beat cycles, relaxation in S11 became progressively impaired, ultimately leading to significant deformation of the heart geometry. To illustrate this effect, Figure 8 compares S11 with the control scenario S00 at the fifth cardiac cycle, showing fiber active stress and fiber stretch at both ED and ES across the basal, equatorial, and apical regions. The equatorial region of S11 appears markedly compressed relative to S00, and ES stress in the basal and equatorial regions is greatly elevated. However, despite the high contractile force, strain changes were minimal, resulting in little color variation in the stretch maps. Examining the radial distance from the basal to the equatorial region shows that, in S00, the thickness between the epicardium and endocardium decreases at ED and increases at ES, whereas in S11 the wall is already thickened at ED. In contrast, when $L_{0}$ increased above 1.58 μm–1.68 μm, the results approached those of the normal case in Figure 8. This also indicates a reduction in SLD, which can be understood by referring to Figure 1b, where the systolic portion of the curve shifts to the right. Even with $L_{r}$ fixed at 1.85 μm, the starting tension is lower, and with lower tension, Figure 2b shows that active stress is reduced. Consequently, relaxation is enhanced, but reduced contractile force leads to less shortening.

#### 3.2.4 Scenario S2 – variable $L_{r}$ , and $L_{0}$ , fixed SLD

The motivation for varying both $L_{r}$ and $L_{0}$ while keeping SLD fixed was to isolate the effect of $L_{r}$ on contraction timing. As demonstrated in Figure 3c, when the SLD is constant, the peak active stress does not change significantly, whereas the contraction time i.e., DOC is strongly influenced by $L_{r}$ . Extending this investigation to the organ level, Figure 9 presents the strain responses under fixed SLD conditions. In this S2 series, $L_{r}$ and $L_{0}$ were co-varied by the same increment to preserve a constant SLD, forming a coupled $L_{r}-L_{0}$ path in parameter space with S21–S22 below S00 and S23–S24 above S00. With the exception of the basal radial strain, nearly all strain measures show an inflection-like trend centered around the control case S00. In the scenario labeled S21, where both $L_{r}$ and $L_{0}$ were reduced in order to preserve the same SLD value as the control, the strain decreased noticeably compared with S00. By contrast, in the scenario labeled S24, where both $L_{r}$ and $L_{0}$ were increased together, the resulting strains remained close to those of S00, although a slight reduction could still be observed. This non-monotonic, U-shaped response around S00 is quantified by piecewise linear regression of APS along the coupled $L_{r}-L_{0}$ series and is summarized in Supplementary Table S1. Specifically, the below-baseline segment S21–S22–S00 and the above-baseline segment S00–S23–S24 exhibit opposite slope directions in several regions, indicating that strain decreases when the coupled parameters deviate from the control toward either lower or higher values. Across the three principal directions of deformation—radial strain, circumferential strain, and longitudinal strain illustrated in Figure 9—the simultaneous decrease of both $L_{r}$ and $L_{0}$ , as in S21, pushed strain values far beyond the healthy reference range. This outcome indicates a profound and pathological reduction in myocardial deformation, underscoring the critical impact of altered sarcomere length parameters on ventricular mechanics.

Figure 10 further clarifies the underlying mechanism. In scenario S21, the stress distribution at ES is relatively higher compared with the control. However, the stretch of fiber direction shows only a very limited reduction, which indicates that there is little additional shortening during systole. This finding is consistent with impaired relaxation and restricted systolic deformation. In contrast, scenario S24 exhibits a stress pattern that is very close to that of S00, with only a slightly reduced degree of relaxation at ED. Taken together, these observations indicate that $L_{0}$ ​ has a stronger influence on diastolic relaxation than $L_{r}$ ​ when SLD is kept constant. A short $L_{0}$ ​, even when paired with a proportionally short $L_{r}$ to maintain the same SLD, can lead to markedly abnormal strain patterns and therefore poses a critical risk to the normal contraction–relaxation dynamics of the heart.

### 3.3 Left heart volume and pressure analysis

**FIGURE 11:**
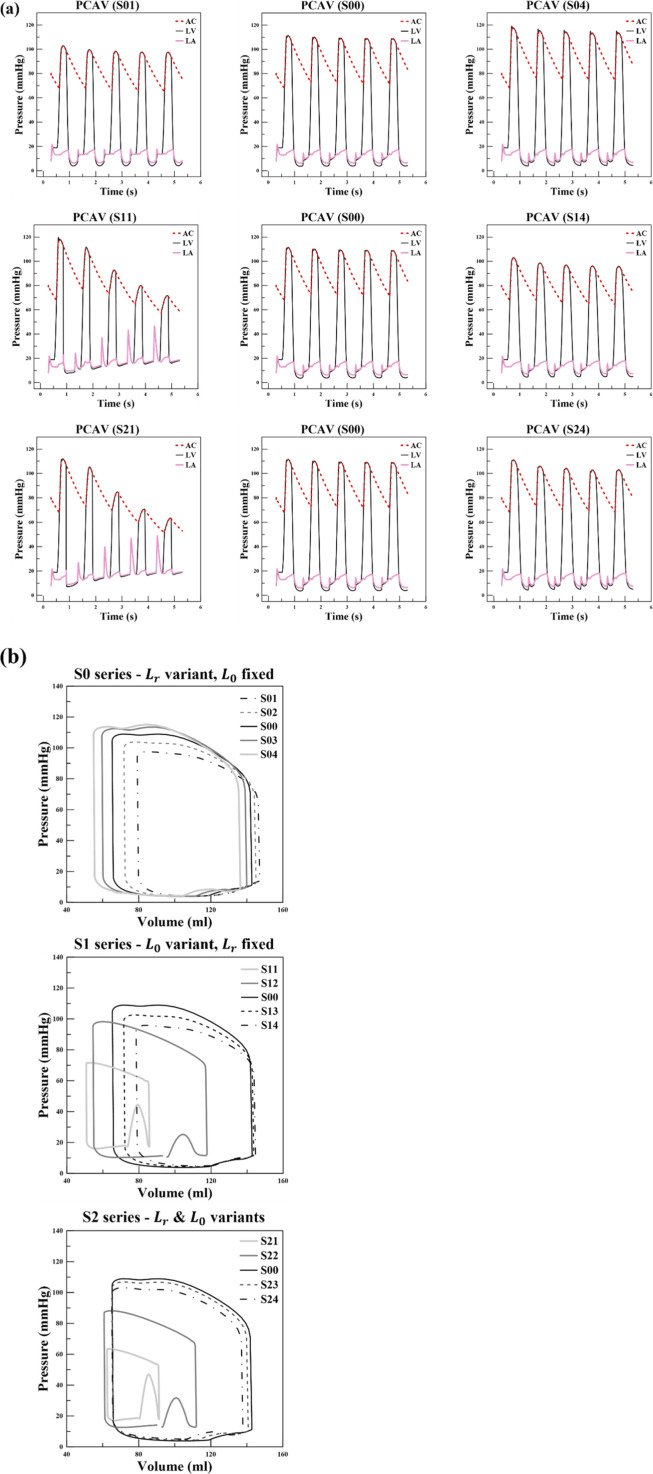
**(a)** Time course of pressures in the left ventricle (LV), left atrium (LA), and arterial compliance compartment (AC) and **(b)** left-ventricular pressure–volume (PV) loops at the fifth cardiac cycle for the S0, S1, and S2 scenario series. The baseline case (S00) is shown in each panel to enable direct within-series comparisons. Legend entries are arranged by increasing SLD; therefore, the S1 cases are listed as S14 → S11 to preserve the same SLD ordering used in the S0 panel (e.g., S01: 0.17 μm → S04: 0.37 μm).

Figure 11a presents the pressure within the fluid cavity (PCAV) and the PV loops for each scenario over time. For comparison, the control case S00 is shown repeatedly in the middle of the plots as a reference. In Figure 11a, which illustrates the pressure variations in the S0 series over time, the pressures of the LV, LA, and arterial compliance (AC) remain steady across five cycles. This indicates that the overall pressure dynamics are stable throughout repeated beats. However, when comparing across different scenarios within the S0 series, both LV and AC pressures show a clear upward trend as $L_{r}$ increases, highlighting that larger $L_{r}$ values are consistently associated with higher pressure generation in these chambers. This scenario-wise pressure increase is consistent with the quantitative changes in global pump performance reported in Table 2 and the sensitivity analysis in Table 3. For example, CO increases from 4.080 L/min in S01 to 4.886 L/min in S04, indicating increased overall pressure-generating capacity at larger $L_{r}$ .

**TABLE 2:** Cardiac function metrics across simulation scenarios, reported as absolute values with percent change relative to the baseline case (S00).

Group	Case	EDV (mL)	ESV (mL)	SV (mL)	LVEF (%)	CO (L/min)
Normal range (Hudsmith et al., 2005; Sievers et al., 2005; Edwards Lifesciences, 2026; Sluysmans and Colan, 2016; Colan, 2016; Yazdi et al., 2021)		102–202	24–92	57–150	50–65	4–8
Baseline	S00 (control)	142.789	65.248	77.541	54.305	4.652
$L_{r}$ series ( $L_{0}$ fixed)	S01	147.147 (+3.05%)	79.149 (+21.30%)	67.998 (−12.31%)	46.211 (−14.90%)	4.080 (−12.30%)
	S02	145.086 (+1.61%)	71.815 (+10.06%)	73.271 (−5.51%)	50.502 (−7.00%)	4.396 (−5.50%)
	S03	139.946 (−1.99%)	59.589 (−8.67%)	80.357 (+3.63%)	57.420 (+5.74%)	4.821 (+3.63%)
	S04	136.392 (−4.48%)	54.962 (−15.76%)	81.430 (+5.02%)	59.703 (+9.94%)	4.886 (+5.03%)
$L_{0}$ series ( $L_{r}$ fixed)	S11	85.827 (−39.89%)	50.745 (−22.23%)	35.082 (−54.76%)	40.875 (−24.73%)	2.105 (−54.75%)
	S12	117.718 (−17.56%)	54.500 (−16.47%)	63.218 (−18.47%)	53.703 (−1.11%)	3.793 (−18.47%)
	S13	143.697 (+0.64%)	71.574 (+9.70%)	72.123 (−6.99%)	50.191 (−7.58%)	4.327 (−6.99%)
	S14	144.654 (+1.31%)	78.371 (+20.11%)	66.283 (−14.52%)	45.822 (−15.62%)	3.977 (−14.51%)
$L_{r}-L_{0}$ combined Series	S21	91.362 (−36.02%)	62.443 (−4.30%)	28.919 (−62.70%)	31.653 (−41.71%)	1.735 (−62.70%)
	S22	112.003 (−21.56%)	60.796 (−6.82%)	51.207 (−33.96%)	45.719 (−15.81%)	3.072 (−33.96%)
	S23	140.746 (−1.43%)	65.127 (−0.19%)	75.619 (−2.48%)	53.727 (−1.06%)	4.537 (−2.47%)
	S24	137.821 (−3.48%)	65.020 (−0.35%)	72.801 (−6.11%)	52.823 (−2.73%)	4.368 (−6.10%)

**TABLE 3:** Sensitivity-based uncertainty analysis of global cardiac metrics across scenario series (baseline: S00).

Metric	Baseline (S00)	( $L_{r}$ ) series: ( $dY/dL_{r}$ )	( $L_{r}$ ) series: ( $S^{*}$ )	$R^{2}$	( $L_{0}$ ) series: ( $dY/dL_{0}$ )	( $L_{0}$ ) series: ( $S^{*}$ )	$R^{2}$	( $L_{r}-L_{0}$ ) series: ( $dY/dL_{r}$ )	( $L_{r}-L_{0}$ ) series: ( $S^{*}$ )	$R^{2}$
EDV (mL)	142.789	−53.3	−0.691	0.987	+287.3	+3.179	0.787	+243.3	+3.153	0.729
ESV (mL)	65.248	−121.2	−3.436	0.992	+144.7	+3.503	0.984	+19.0	+0.538	0.556
SV (mL)	77.541	+67.9	+1.620	0.943	+142.6	+2.906	0.469	+224.4	+5.353	0.718
LVEF (%)	54.305	+67.8	+2.310	0.986	+12.8	+0.371	0.032	+100.7	+3.430	0.690
CO (L/min)	4.652	+4.1	+1.620	0.943	+8.6	+2.906	0.469	+13.5	+5.354	0.719

(1) Global slopes ( dY/dp ) were obtained by linear regression using the five scenarios in each series. (2) Normalized sensitivity was computed as $S^{*}=\left(p_{base}/Y_{base}\right)\left(dY/dp\right)$ , where $p_{base}$ and $Y_{base}$ are the parameter and output values at S00. (3) For the $L_{r}-L_{0}$ series, $dY/dL_{r}$ represents the combined response along the diagonal path where $L_{r}$ and $L_{0}$ increase together.

In contrast, the S1 series, which involves changes in $L_{0}$ , demonstrates more complex behavior than the relatively straightforward S0 series. When $L_{0}$ is increased, as in scenario S14 where the SLD becomes smaller, the time–pressure relation closely resembles that of scenario S01, suggesting that similar mechanisms are involved. However, the behavior diverges markedly in scenario S11. In this case, as the cardiac cycles progress, the pressures in both the LV and the AC decrease significantly, while the pressure in the LA continues to rise, indicating impaired relaxation and abnormal filling dynamics. These abnormalities are reflected quantitatively in Table 2, where S11 shows large reductions in EDV and SV relative to S00. A similar pattern is also evident in the S2 series. For instance, in scenario S24 where $L_{0}$ is lengthened, the pressures remain stable over five cycles and differ little from the control scenario S00, reflecting near-normal behavior. In scenario S21, by contrast, the LV and aortic cavity pressures fall dramatically, while the LA pressure progressively increases, highlighting once again the detrimental effect of a shortened $L_{0}$ on overall hemodynamics. Table 2 further supports this interpretation by showing that S21 exhibits severe reductions in SV and CO compared with S00, consistent with the collapsing pressure dynamics observed over repeated cycles.

Figure 11b shows the PV loops after five cycles. In the S0 series, a clear trend emerges: when $L_{r}$ decreases, EDV becomes larger and ESV also increases, whereas when $L_{r}$ increases, both EDV and ESV are reduced. However, as shown in Table 2, SV, LVEF, and CO all increase with larger $L_{r}$ . SV refers to the volume of blood ejected by the ventricle per heartbeat and is a standard metric for assessing cardiac output i.e., CO (Hayabuchi et al., 2023; Singh et al., 2021; Seemann et al., 2023). LVEF represents the percentage of blood pumped out of the ventricle during systole and is considered a key clinical indicator of cardiac function. CO reflects the total blood volume pumped per minute (Konstam and Abboud, 2017; Al Younis et al., 2024). In this study, the durations of both systolic contraction (beat) and diastolic relaxation (recovery) were set to 0.5 s, resulting in a total cycle length of 1 s. Accordingly, the model simulates 60 cardiac cycles per minute, and CO was calculated on this basis. These findings are consistent with the strain and stress results presented in Figures 4, 5. When $L_{r}$ begins from a shorter value, the myocardium is initially under less tension, leading to better relaxation, but the weaker contractile force results in reduced systolic shortening. This behavior is directly reflected in larger EDV and ESV. Conversely, when $L_{r}$ is longer, the myocardium undergoes less diastolic relaxation, resulting in reduced EDV, but the higher contractile force produces a markedly smaller ESV. Consequently, the difference between EDV and ESV, and therefore SV, LVEF, and CO, all increase. These patterns are characteristic of inotropic modulation, and the results strongly suggest that $L_{r}$ is directly related to inotropic function. To quantify this dependence, Table 3 reports global slopes and normalized sensitivities with respect to $L_{r}$ , providing a quantitative measure of how EDV, ESV, SV, LVEF, and CO change across the S0 series.

Unlike the S0 series, which exhibited a relatively clear and consistent pattern, the S1 series showed far more complex PV loop dynamics due to changes in $L_{0}$ . When $L_{0}$ was increased beyond the baseline value of 1.58 μm, as in scenarios S13 and S14, the PV loops showed only a very slight increase in EDV along with a modest rise in ESV. These results closely resembled the transition observed between S01 and S00 in the S0 series, indicating that the overall behavior could be interpreted as a manifestation of reduced inotropy. This relatively mild above-baseline response is quantified by the piecewise sensitivity analysis in Table 4 for the segment S00–S13–S14, which yields small slopes for several PV-derived metrics compared with the below-baseline segment. However, when $L_{0}$ was shortened below the baseline, as in scenario S11, the situation changed dramatically. The time-dependent pressure profiles collapsed, and the PV loop became markedly smaller, with EDV reduced substantially and ESV also diminished. As a direct consequence of these reductions, SV, LVEF, and CO all decreased significantly, confirming that shortening $L_{0}$ impairs both relaxation and pumping efficiency. As reported in Table 2, EDV decreases from 142.789 mL in S00 to 85.827 mL in S11, while SV decreases from 77.541 mL to 35.082 mL, demonstrating a severe loss of pumping capacity when $L_{0}$ ​ is shortened below baseline. The S2 series, evaluated relative to the control scenario S00, revealed a similar tendency but with even more pronounced abnormalities. When both $L_{0}$ and $L_{r}$ were increased together, as in S24, ESV remained nearly unchanged and EDV showed only a gradual decrease, indicating relatively stable function. By contrast, when $L_{0}$ was shortened, as in S21 and S22, the effects were severe. In S21, EDV dropped drastically while ESV showed little variation, leading to a dramatic reduction in SV, LVEF, and CO. In S22, the magnitude of the changes was less extreme but the overall trend was the same: LV and AC pressures progressively declined with each cycle, LA pressure steadily increased, and the PV loop revealed a marked reduction in EDV. Table 2 further emphasizes these findings. Values highlighted in red denote results falling outside the normal physiological range, and many such abnormalities were observed in scenarios where $L_{0}$ was shortened. This indicates that reducing $L_{0}$ below the control value can exert a critical and pathological influence on cardiac function. This asymmetry around S00 is quantified by the piecewise analysis in Table 4, which shows substantially larger sensitivities in the below-baseline segments compared with the above-baseline segments, consistent with the pronounced pathological deterioration observed for S11 and S21.

**TABLE 4:** Piecewise sensitivity analysis around the baseline (S00) for $L_{0}$ and the $L_{r}-L_{0}$ .

Metric	S11–S12–S00: ( $dY/dL_{0}$ )	$S^{*}$	$R^{2}$	S00–S13–S14: ( $dY/dL_{0}$ )	$S^{*}$	$R^{2}$	S21–S22–S00: (d $dY/dL_{r}$ )	$S^{*}$	$R^{2}$	S00–S23–S24: ( $dY/dL_{r}$ )	$S^{*}$	$R^{2}$
EDV (mL)	+569.6 mL/μm	+6.303	0.995	+18.7 mL/μm	+0.206	1.000	+514.3 mL/μm	+6.663	0.987	−49.7 mL/μm	−0.644	0.990
ESV (mL)	+145.0 mL/μm	+3.512	0.928	+131.2 mL/μm	+3.178	1.000	+28.1 mL/μm	+0.795	0.388	−2.3 mL/μm	−0.065	0.999
SV (mL)	+424.6 mL/μm	+8.652	0.966	−112.6 mL/μm	−2.294	1.000	+486.2 mL/μm	+11.600	0.998	−47.4 mL/μm	−1.131	0.988
LVEF (%)	+134.3%/μm	+3.907	0.784	−84.8%/μm	−2.468	1.000	+226.5%/μm	+7.717	0.981	−14.8%/μm	−0.505	0.984
CO (L/min)	+25.5 L/min/μm	+8.651	0.966	−6.8 L/min/μm	−2.293	1.000	+29.2 L/min/μm	+11.600	0.998	−2.8 L/min/μm	−1.129	0.988

(1) Slopes ( dY/dp ) were obtained by linear regression using the three scenarios in each segment. (2) Normalized sensitivity $S^{*}=\left(p_{base}/Y_{base}\right)\left(dY/dp\right)$ , with baseline values from S00. (3) For the $L_{r}-L_{0}$ , $dY/dL_{r}$ represents the combined response along the diagonal path where $L_{r}$ and $L_{0}$ increase together.

**FIGURE 12:**
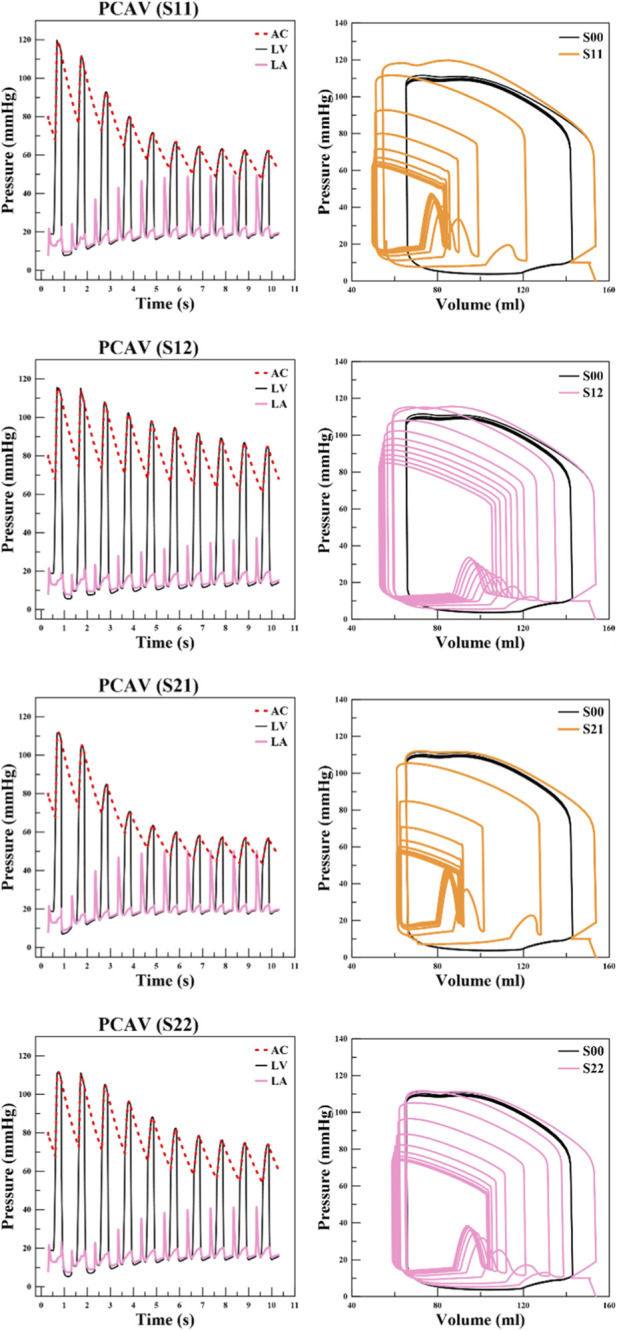
Time course of pressures in the left ventricle (LV), left atrium (LA), and arterial compliance compartment (AC), and the corresponding left-ventricular pressure–volume (PV) loops over 10 cardiac cycles for scenarios S11, S12, S21, and S22.

For both the S1 and S2 series, a shortened $L_{0}$ caused the PV loops to fail to close properly and to contract progressively over successive cycles. To verify these observations, extended simulations were performed over ten cardiac cycles, with pressure and volume changes monitored in detail. The results are summarized in Figure 12. In S11, during the early cycles, LV and AC pressures declined sharply while LA pressure increased continuously. After approximately five cycles, the system began to stabilize, but the PV loop still showed clear differences compared with the control S00. EDV progressively decreased from cycle to cycle, while ESV remained almost unchanged. In S12, where $L_{0}$ was shortened to a lesser extent, the cycle-to-cycle pressure changes were less dramatic, yet EDV continued to decline gradually, with ESV remaining nearly constant. The outcomes in the S2 series were even more severe. In S21, LV and AC pressures fell sharply as the cycles progressed, while LA pressure rose significantly. By the sixth cycle, the peak pressure in the LA even exceeded that in the AC, a severely abnormal phenomenon not observed in the control. The PV loop in S21 revealed a striking reduction in EDV. In S22, the abnormalities were less extreme but still pronounced. Again, LV and AC pressures declined cycle after cycle, LA pressure steadily increased, and the PV loop demonstrated a significant reduction in EDV. Together, Tables 2–4 provide quantitative support for these observations by reporting effect sizes relative to S00 and sensitivity metrics that identify shortened $L_{0}$ regimes as highly pathological and strongly output-sensitive.

### 3.4 Cross-scenario ranking based on deviations from normal ranges.

**FIGURE 13:**
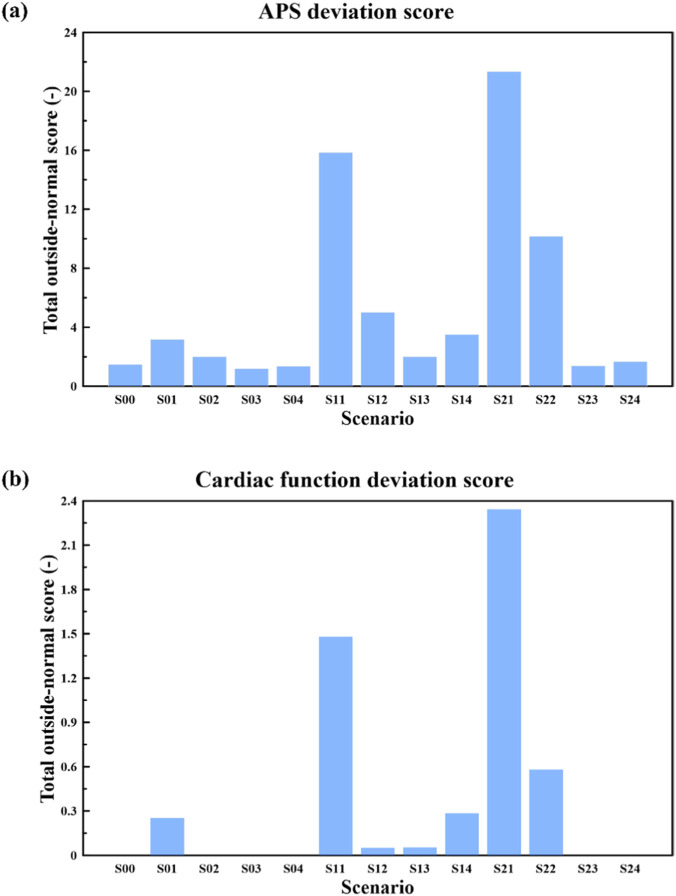
Cross-scenario rankings based on deviations from literature-reported normal ranges at the fifth cardiac cycle: **(a)** APS deviation score. Scenarios were ranked using the summed deviation score across average peak strain (APS) metrics relative to literature-reported mean ± SD (Figures 4, 6, 9). **(b)** Cardiac function deviation score. Scenarios were ranked using the summed deviation score across global cardiac function metrics (EDV, ESV, SV, LVEF, and CO) relative to reported normal ranges (Table 2). **(b)**Deviation-score definitions are provided in Section 3.4.

To facilitate cross-scenario comparison and identify the most abnormal cases, we computed deviation scores relative to literature-reported normal ranges at the fifth cardiac cycle and summarized scenario rankings in Figure 13. For APS, the outside-normal score was defined using the literature mean μ and standard deviation σ (Figures 4, 6, 9), as given in Equation 15,

$$d_{APS}=\max\left(0,\left|\frac{x-\mu }{\sigma }\right|-1\right),$$ (15)

where $d_{APS}=0$ indicates x lies within μ±σ . The APS deviation score (Figure 13a was obtained by summing $d_{APS}$ across APS metrics.

For cardiac function metrics, for each metric value x with normal range $\left[L,U\right]$ (Table 2), the outside-normal score was defined as

$$d_{func}=\max\left(0,\frac{L-x}{U-L}\right)+\max\left(0,\frac{x-U}{U-L}\right)$$ (16)

where $d_{func}=0$ indicates $x\in \left[L,U\right]$ . The cardiac-function deviation score (Figure 13b) was obtained by summing $d_{func}$ across EDV, ESV, SV, LVEF, and CO.

## 4 Discussions

In this study, the LHM was employed to investigate how variations in SL, specifically the initial reference length $L_{r}$ and the threshold length for active contraction $L_{0}$ , affect myocardial tissue mechanics and overall cardiac function. The PV loops of LV are provided in Figure 11b and Figure 12 and are referenced throughout the Discussion. The analysis revealed that $L_{r}$ primarily acts as a determinant of inotropy, whereas $L_{0}$ , when shortened below the baseline value of 1.58 μm, induces pronounced diastolic dysfunction. Changes in $L_{0}$ demonstrated two distinct effects depending on whether the value was above or below the baseline of 1.58 μm. When $L_{0}$ increased above this threshold, inotropy was reduced, mirroring the results observed in the S0 series when $L_{r}$ was decreased; in both cases, the reduction in SLD led to diminished contractile strength. However, when $L_{0}$ was shortened below 1.58 μm, a pronounced deterioration in hemodynamics was observed. As summarized in Table 2, EDV decreased from 142.789 mL to 85.827 mL in S11 (−39.9%). The cycle-to-cycle pressure patterns indicate that the heart failed to relax adequately during diastole, resulting in insufficient preload. In addition, contractile force was impaired, further limiting systolic ejection. Consequently, SV decreased from 77.541 mL to 35.082 mL in S11 (−54.8%).

When $L_{0}$ is maintained at the normal reference value of 1.58 μm, increasing $L_{r}$ produces a physiological pattern characterized by elevated end-systolic pressure (ESP), reductions in both EDV and ESV, and a corresponding increase in SV. In addition, the slope of the end-systolic pressure–volume relationship (ESPVR) becomes steeper (van der Velden and Stienen, 2019). These features resemble conditions associated with enhanced inotropy, such as heightened sympathetic stimulation, catecholamine excess, the administration of positive inotropic agents, or the early compensatory stage of heart failure (Klabunde, 2021a). By contrast, decreasing $L_{r}$ evokes a pattern consistent with impaired contractility. This resembles the phenotype of heart failure with reduced ejection fraction (HFrEF), as typically seen in dilated cardiomyopathy, post-ischemic or post-infarction remodeling, or in response to the use of negative inotropic drugs (Klabunde, 2021a; Hamo et al., 2024).

In contrast to the effects of varying $L_{r}$ , the S1 series, which explores changes in $L_{0}$ , reveals two distinct patterns depending on whether $L_{0}$ is increased or decreased relative to the baseline value of 1.58 μm. When $L_{0}$ is lengthened beyond the baseline, EDV remains nearly unchanged, whereas ESV shows a progressive increase, suggesting a reduction in inotropy. Consistent with this interpretation, SV and LVEF also exhibit a decreasing trend in these cases. Overall, this response is similar to that observed when $L_{r}$ is reduced in the S0 series, suggesting that, in the high- $L_{0}$ regime, the global pump behavior is influenced predominantly by changes in SLD (Klabunde and Klabunde, 2013; Klabunde, 2021b). Under these conditions, ventricular relaxation is slightly enhanced, while the reduced contractile performance inferred from the increases in ESV and the decreases in SV and LVEF yields trends similar to those observed when $L_{r}$ is reduced in the S0 series. This suggests that at higher values of $L_{0}$ , the role of SLD becomes particularly important, as it not only determines the initial position on the length–tension curve but also governs the peak active stress of myocardial tissue, making it a parameter that warrants close attention. The critical issue arises when $L_{0}$ is shortened below the baseline value of 1.58 μm. In this case, markedly adverse changes occur, most notably a severe reduction in EDV. This phenomenon appears to stem from a fundamental alteration of the length–tension relationship: at any given sarcomere length, tension begins at a much higher level, thereby lowering diastolic compliance. The ventricle consequently becomes stiffer, requiring higher filling pressures to achieve the same volume. As reflected in the PV loops of scenarios S11 and S12, shortening $L_{0}$ produced a leftward shift of the loop with reduced EDV and ESV and a smaller loop area, which qualitatively resembles the hemodynamic pattern reported in mitral stenosis, where restricted transmitral inflow limits ventricular filling and results in reduced chamber volumes and diminished PV-loop area. (Neema, 2015; Motshabi-Chakane, 2023). Although these low- $L_{0}$ cases exhibited non-periodic transient behavior during the initial beats, the pronounced cycle-to-cycle drift decreased after several beats, and the later-beat PV loops show a consistently reduced filling state. Under otherwise identical LHM settings, this behavior suggests that lowering $L_{0}$ can mechanically impede ventricular filling and strongly perturb diastolic function and atrial–ventricular–circulatory coupling. In the TVE formulation $L_{0}$ is often viewed primarily as a parameter that modulates ${Ca}^{2+}$ sensitivity. However, our results indicate that changing $L_{0}$ also shifts the effective length–tension relationship and can substantially affect both myocardial mechanics and chamber-level function, warranting further investigation into its physiological significance.

In the S2 series, we examined the combined effects of varying both $L_{r}$ and $L_{0}$ while keeping SLD constant. As a result, the peak active stress at the material level was essentially preserved, and the main change across scenarios was the duration of contraction. When both $L_{r}$ and $L_{0}$ were increased simultaneously, such that $L_{0}$ ​ exceeded the baseline value of 1.58 μm, ESV remained nearly unchanged whereas EDV decreased, yielding a pattern that resembles reduced preload, in which impaired filling lowers EDV while systolic emptying capacity is largely maintained (Klabunde, 2021b; Kirkman, 2018). By contrast, when both $L_{r}$ and $L_{0}$ were decreased such that $L_{0}$ fell below 1.58 μm, EDV dropped abnormally and filling pressure failed to develop adequately, consistent with the severe diastolic-filling impairment observed in the S1 series at low $L_{0}$ .

In scenarios S11, S12, S21, and S22, where $L_{0}$ was reduced below its baseline value, we observed pronounced abnormalities in the pressure waveforms as shown in Figure 12. In these low- $L_{0}$ cases, global LV pump function was reduced, as reflected by lower LV pressure and LVEF (Leite-Moreira et al., 2012). In addition, LA pressure transiently exceeded LV pressure across these cases and, in the extreme case of scenario S21, also exceeded the AC pressure. To interpret these pressure patterns, it is useful to recall how pressures arise in the LHM. In the present simulations, LV/LA pressures are not imposed *a priori*; instead, they emerge from the coupled deformation of the chambers with the closed-loop lumped-parameter circulation, such that changes in ventricular contractile performance directly influence the time-varying LV pressure waveform. In our low- $L_{0}$ cases, the reduction in LVEF was accompanied by an overall reduction in LV pressure, which is qualitatively consistent with reports that pressure generation and diastolic pressure responses depend on systolic function, particularly in patients with moderately depressed ejection fraction (approximately 30%–49%) (Leite-Moreira et al., 2012) Under such conditions, impaired LV filling and reduced suction can require a larger atrial-to-ventricular pressure gradient to drive transmitral inflow; consequently, LA pressure can rise and exhibit a spiky waveform (Fukuta and Little, 2008). At the same time, the episodes in which LA pressure transiently exceeded LV pressure (and, in scenario S21, even exceeded AC pressure) should be viewed as nonstandard behavior that would be rare *in vivo*. Because LA/PCAV and AC pressures are obtained through the coupled fluid-cavity and lumped-parameter circulation formulations, these extreme excursions may reflect transient coupling effects or sensitivity to parameter tuning in extreme regimes. Nevertheless, the consistent appearance of abnormal LA/PCAV pressure patterns and impaired filling under otherwise identical LHM settings reinforces the conclusion that decreasing $L_{0}$ can strongly perturb diastolic filling and atrial–ventricular–circulatory coupling. Taken together, these observations suggest that decreasing $L_{0}$ can drive highly abnormal organ-level behavior within the LHM framework, even though the most extreme excursions should be interpreted cautiously. Such changes are associated with profoundly abnormal cardiac responses, often more striking than those produced by variations in $L_{r}$ . Unlike $L_{r}$ , which represents an actual measurable sarcomere length, $L_{0}$ is derived theoretically by subtracting passive tension from total tension to define the threshold for active contraction. Because it is not a directly measurable parameter, $L_{0}$ has historically received little attention, and even state-of-the-art platforms such as the Living Heart Model continue to rely on the decades-old value reported by ter Keurs et al. (1980). Despite this limitation, the present results demonstrate that $L_{0}$ remains a critical determinant of myocardial behavior, reshaping the entire length–tension relationship and thereby warranting deeper investigation.

Notably, the deviation-score rankings in Figure 13 suggest that scenarios S11 and S21 are among the most deviating cases relative to literature-reported normal ranges, based on both average peak strain and global cardiac function. These scenarios share the common feature that $L_{0}$ is reduced to 1.48 μm, which is 0.1 μm below the baseline value of 1.58 μm, indicating that even modest shortening of $L_{0}$ can be associated with disproportionately abnormal organ-level responses. This observation supports the view that $L_{0}$ , often treated as a theoretical parameter in the TVE formulation, should also be considered in disease-oriented investigations and in future efforts to refine and calibrate computational heart models.

From a clinical perspective, these findings highlight the importance of considering $L_{0}$ not only as a theoretical construct but as a potential contributor to both congenital and acquired cardiac disorders. Alterations in $L_{0}$ may be linked to genetic variants in proteins related to sarcomeres, as well as to secondary changes due to myocardial infarction, remodeling, or protein degeneration (Linke, 2018; Tanner et al., 2023). Thus, bridging sarcomere mechanics with clinical cardiology could provide new insights into the pathophysiology of diastolic dysfunction, cardiomyopathy, or valvular disease (van Heerebeek et al., 2006; Garfinkel et al., 2018; Solaro et al., 2024). Looking ahead, future research should expand beyond sarcomere length alone to include parameters related to the velocity of length change, ${Ca}^{2+}$ concentration dynamics, and other modulators of excitation–contraction coupling (Mojumder et al., 2023; Motchon et al., 2024). Such studies could leverage the Living Heart Model to develop disease-specific models and even explore aimed at correcting the abnormal responses observed in this work (Reza et al., 2022; Katsoulakis et al., 2024). At the same time, it must be acknowledged that this model is inherently limited by its simulation-based nature. Because $L_{0}$ is difficult to manipulate or validate experimentally, the scenarios tested here remain somewhat arbitrary and cannot fully replicate human physiology. Nevertheless, our results show that variations in sarcomere-related parameters within the Living Heart framework can substantially alter contractile behavior, and we hope this work provides a modest contribution to refining computational cardiac models toward greater physiological relevance. Still, if cardiac simulations are continuously refined and validated, they may eventually evolve into powerful tools that surpass experimental limitations and deliver clinically reliable insights (Samant et al., 2023; Zhou et al., 2024; Baylous et al., 2024).

Taken together, the present study suggests that variations in $L_{0}$ can provoke far more severe abnormalities than variations in $L_{r}$ , a novel finding that underscores the necessity of integrating sarcomere-level mechanics, organ-level hemodynamics, and clinical pathology into a unified framework.

## 5 Conclusion

This study systematically evaluated the mechanistic impact of two key sarcomere length parameters on myocardial contraction dynamics and global left heart function using the Living Heart Model framework. The first parameter, $L_{r}$ , is defined as the initial or unloaded sarcomere length, representing the baseline length before contraction begins. The second parameter, $L_{0}$ , is defined as the sarcomere length at which no active tension develops, serving as the threshold for the onset of active force generation.

At the tissue scale, $L_{r}$ primarily modulated contraction timing and inotropic response, whereas $L_{0}$ governed both peak active stress and diastolic relaxation by shifting the length–tension curve. The SLD emerged as a unifying determinant of peak stress, reinforcing its role as a central parameter in myocardial mechanics. At the organ scale, increasing or decreasing $L_{r}$ altered inotropy in a manner that produced predictable changes in EDV, ESV, and PV loop morphology. The present findings suggest that excessively short or long $L_{r}$ values would ultimately manifest as patterns reflecting alterations in inotropy. By contrast, variations in $L_{0}$ caused much more pronounced abnormalities. While elevated $L_{0}$ promoted relaxation but reduced contractile force, reduced $L_{0}$ produced striking diastolic dysfunction, preload reduction, and PV loop collapse. These behaviors resembled impaired ventricular filling and abnormal pressure–volume dynamics similar to those seen in obstructive or restrictive conditions, though no direct clinical equivalence can be claimed from the present simulations.

Given the magnitude of these effects, further *in silico* and experimental studies focusing on $L_{0}$ modulation, are warranted. Such studies should aim both to elucidate the underlying molecular drivers, including shifts in titin isoforms and mutations of myofilament proteins, and to explore potential therapeutic strategies for restoring optimal sarcomere operating ranges. Ultimately, $L_{r}$ and $L_{0}$ represent not only key biophysical determinants of myocardial performance but also promising diagnostic and prognostic biomarkers for disorders associated with abnormal sarcomere function.

## Data Availability

The original contributions presented in the study are included in the article/Supplementary Material, further inquiries can be directed to the corresponding author.

## Glossary

$\Psi_{\text{dev}}$: deviatoric component of the strain-energy potential
$\Psi_{\text{dev}}^{i}$: isotropic component of the deviatoric strain-energy potential
$\Psi_{\text{dev}}^{t}$: transversely isotropic component of the deviatoric strain-energy potential
$\Psi_{\text{dev}}^{o}$: orthotropic component of the deviatoric strain-energy potential
$\Psi_{\text{vol}}$: volumetric component of the strain-energy potential
**()**_**f**_: fiber direction
**()**_**s**_: sheet direction
$a,b,a_{f},b_{f},a_{s},b_{s},a_{\text{fs}},b_{\text{fs}}$: material constants
$I_{1}$: first deviatoric strain invariant
$I_{4f},I_{4s}$: pseudo-invariants for the transversely isotropic component ( $=A_{f}\cdot C\cdot A_{f}$ or $A_{s}\cdot C\cdot A_{s}$ )
$I_{8\text{fs}}$: pseudo-invariant for the orthotropic component ( $=A_{f}\cdot C\cdot A_{s}$ )
C: right Cauchy-Green deformation tensor
$A_{f},A_{s}$: fiber and sheet direction vectors
D: multiple of bulk modulus ( K=2/D )
J: third invariant of the deformation gradient (Jacobian)
$\sigma_{f}$: total stress in the fiber direction
$\sigma_{\text{pf}}$: passive stress
$\sigma_{\text{af}}$: active stress
$T_{\max}$: maximum isometric tensile stress achieved at the longest sarcomere length
$Ca_{0}$: the peak intracellular calcium ( $\left.{Ca}^{2+}\right)$ concentration
${\left(Ca_{0}\right)}_{\max}$: the maximum intercellular calcium ( ${Ca}^{2+}$ ) concentration
$B,m,b^{\prime }$: material constants
$l_{0}$: the sarcomere length below which no active force develops
$t_{0}$: time to reach the peak tension
$l_{r}$: initial sarcomere length
$E_{\text{ff}}$: fiber-direction strain
$\sigma_{ff}$: Cauchy stress component in the fiber direction (fiber–fiber component)
$\sigma_{ss}$: Cauchy stress component in the sheet direction (sheet–sheet component)
$\lambda_{f}$: principal stretch ratio in the fiber direction
$\lambda_{s}$: principal stretch ratio in the sheet direction
$\lambda_{n}$: principal stretch ratio in the sheet-normal (normal) direction
e: nominal (engineering) strain
