# Supplementary figures and images for: In silico analysis of sarcomere length effects on myocardial contraction and cardiac function using the living left heart model
Source: Front Bioeng Biotechnol. 2026 Apr 10;14:1756525. doi: 10.3389/fbioe.2026.1756525 (PMC13106318; doi:10.3389/fbioe.2026.1756525)

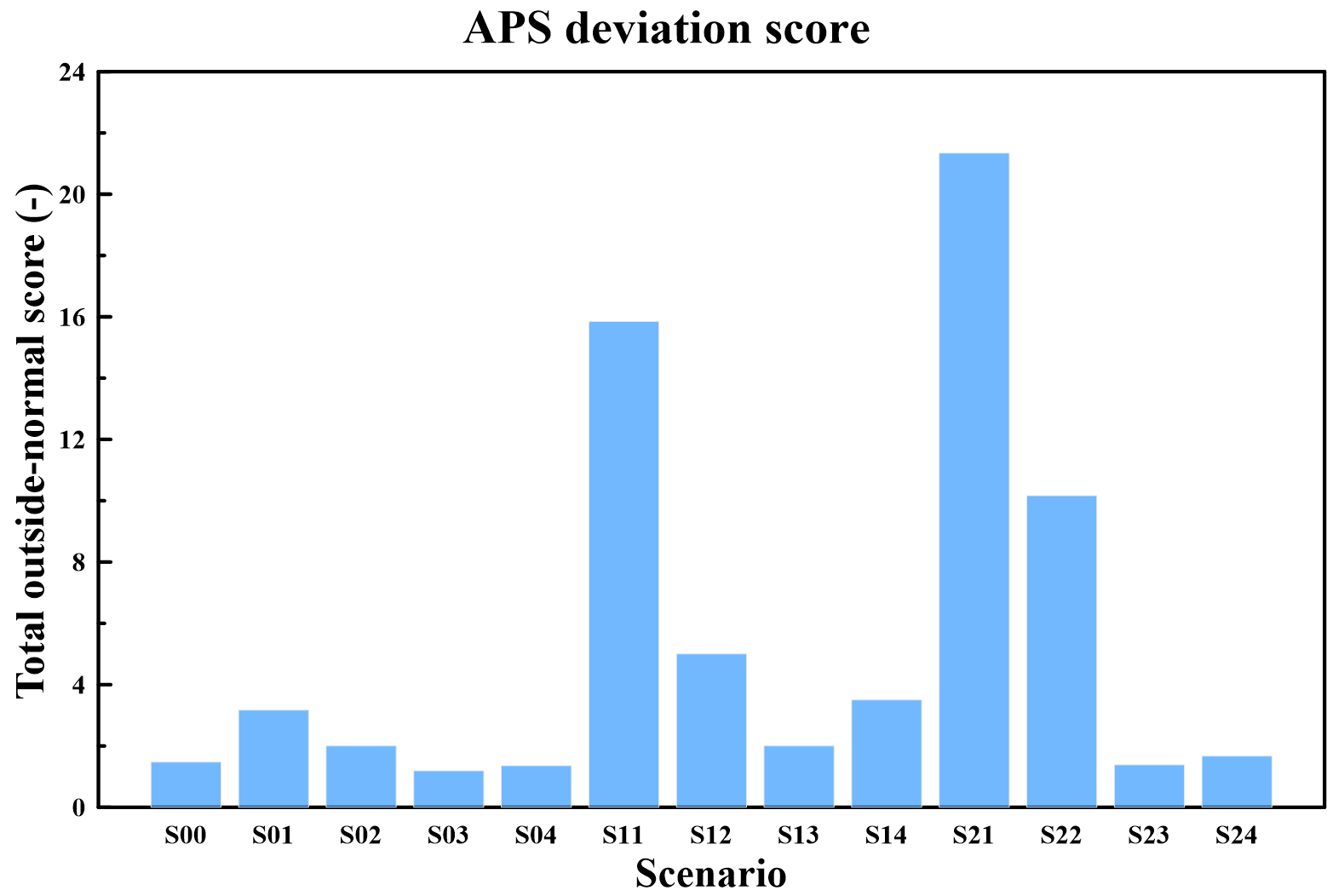

Supplement: Supplementary file 1 [file Image6.tif]

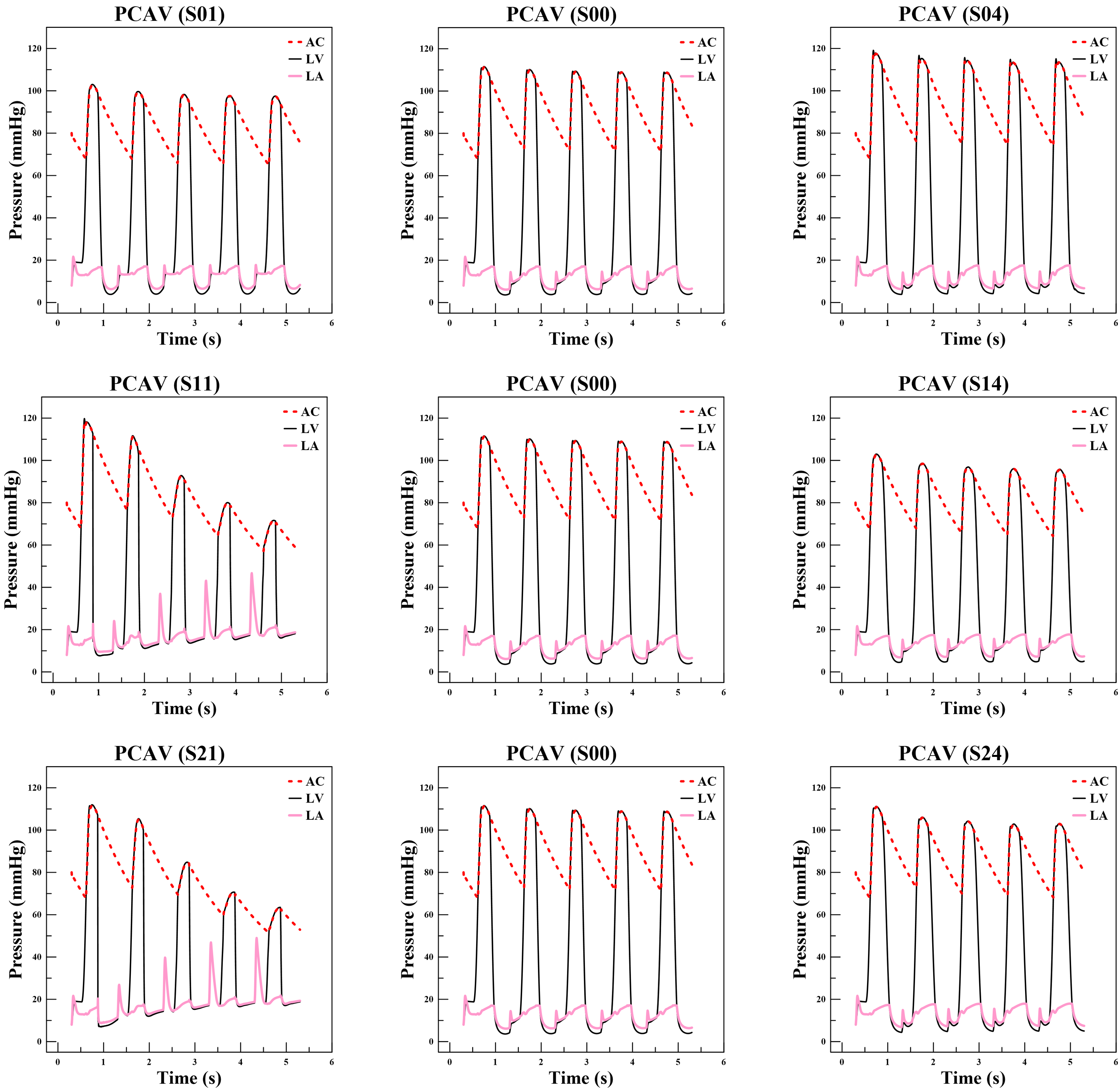

Supplement: Supplementary file 3 [file Image3.tif]

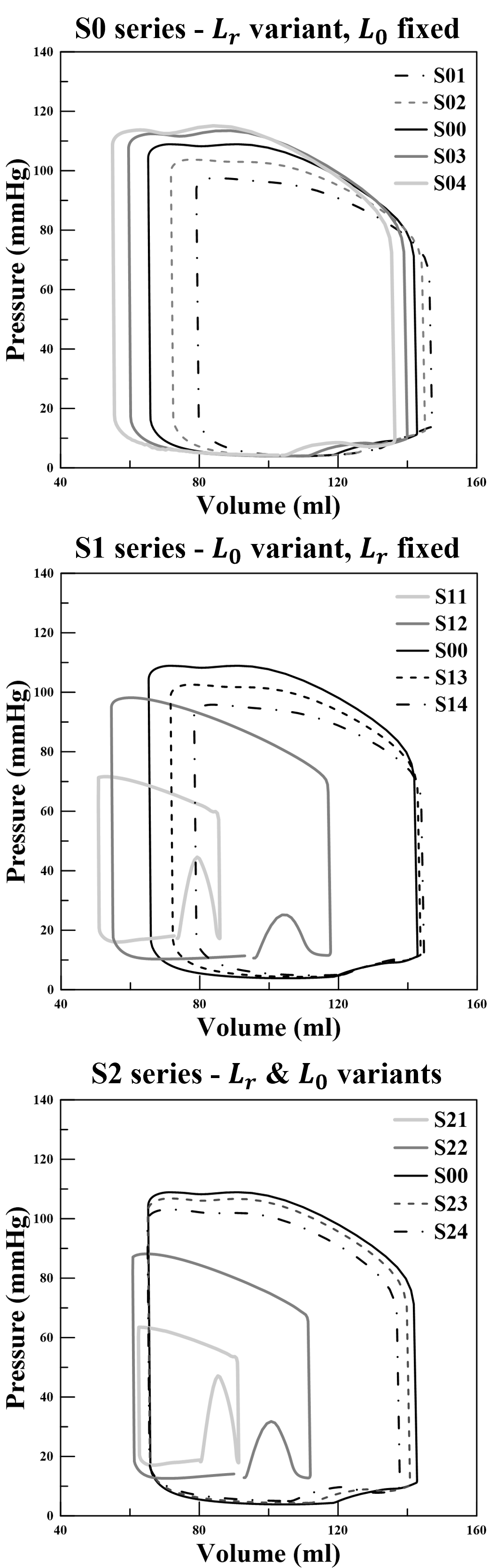

Supplement: Supplementary file 4 [file Image4.tif]

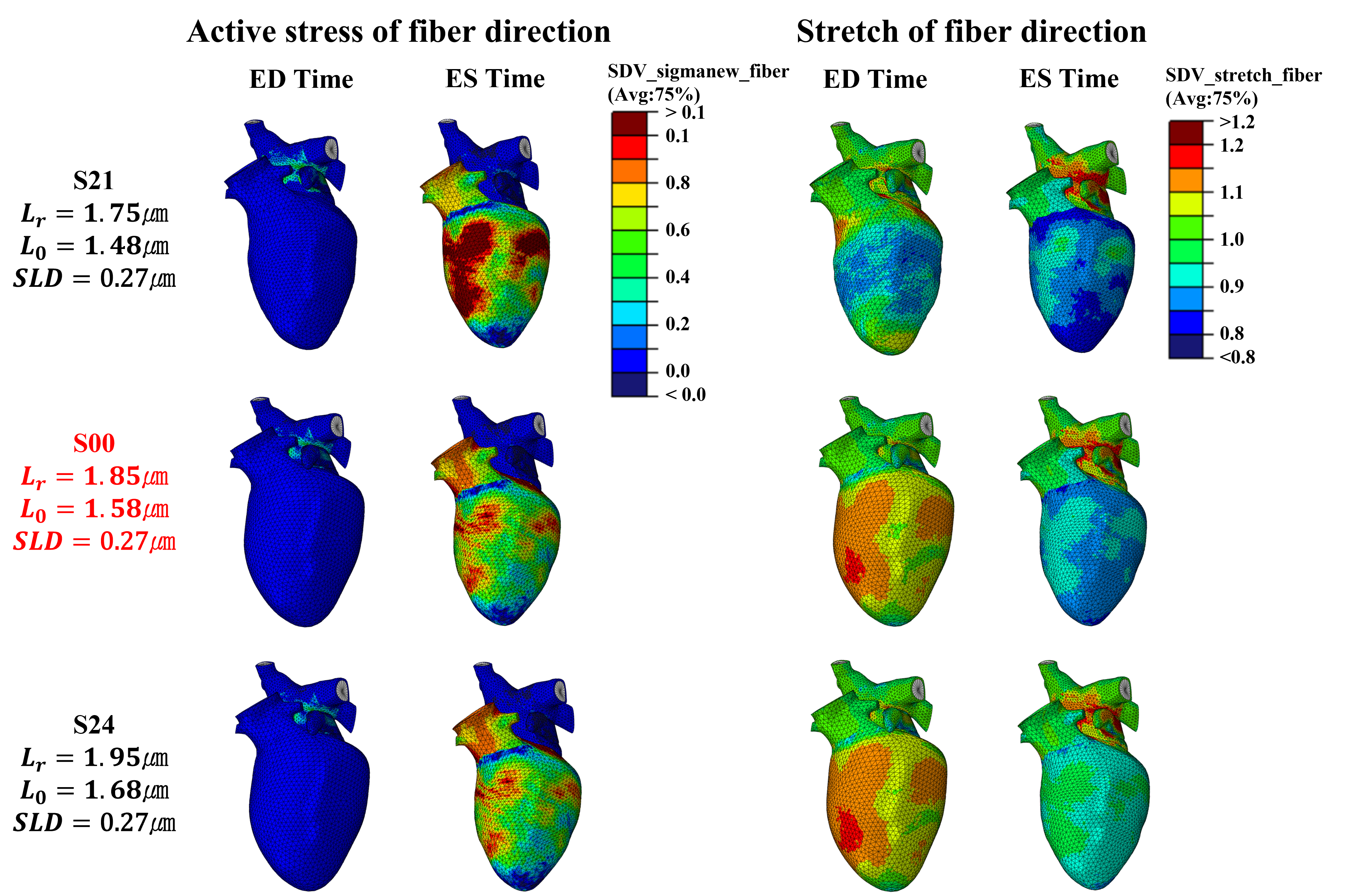

Supplement: Supplementary file 5 [file Image2.tif]

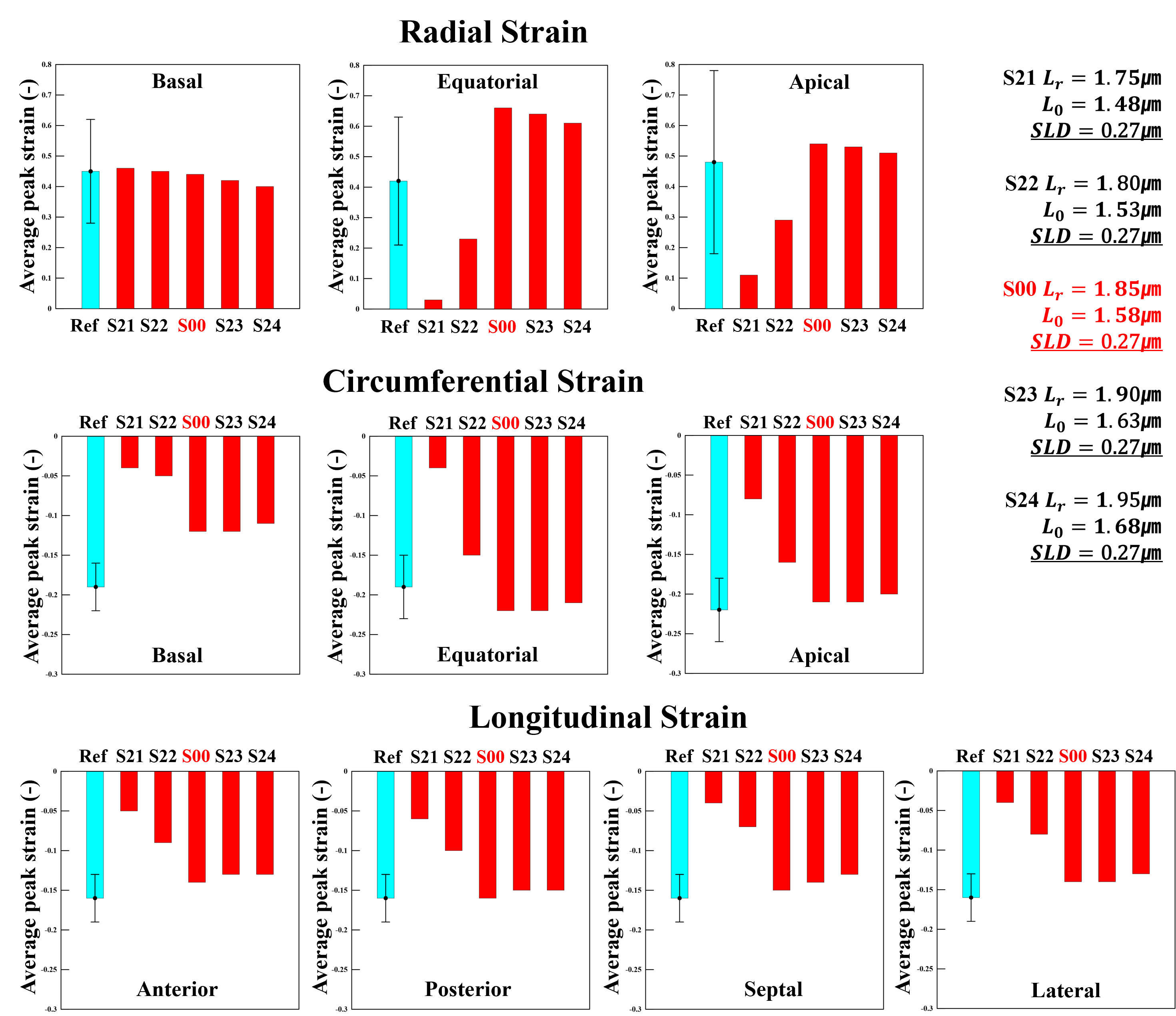

Supplement: Supplementary file 6 [file Image1.tif]

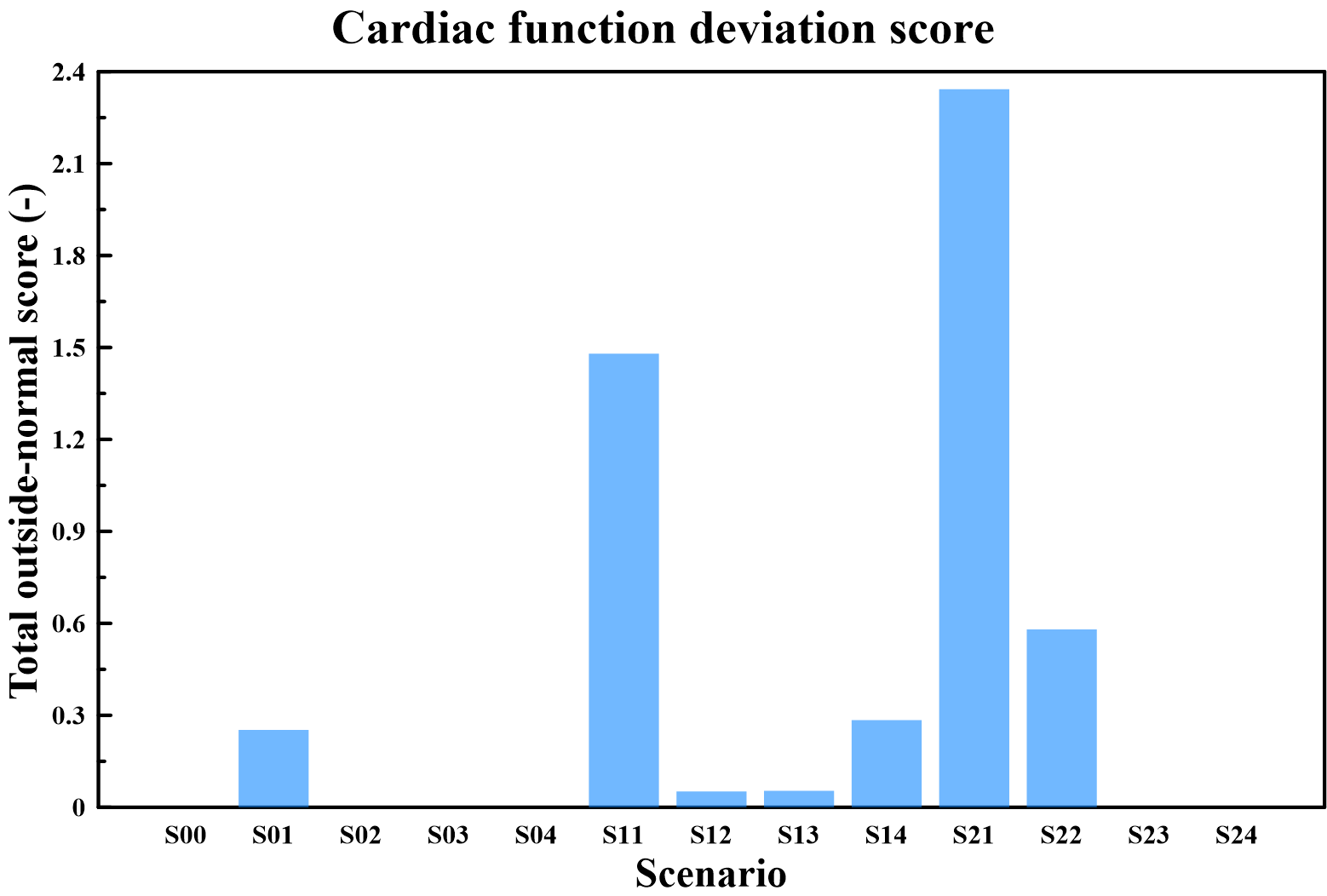

Supplement: Supplementary file 7 [file Image7.tif]

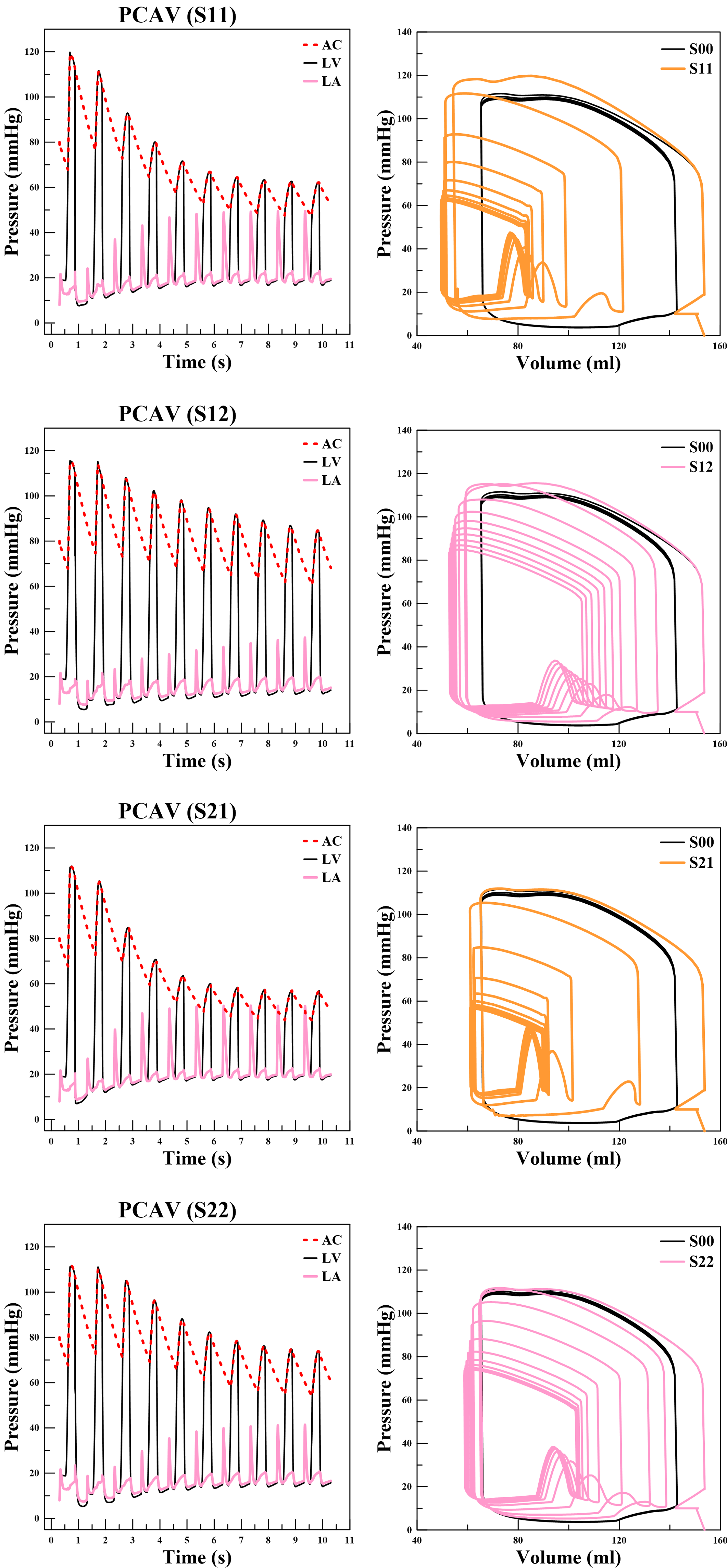

Supplement: Supplementary file 8 [file Image5.tif]
