# Supplementary material for: In silico analysis of sarcomere length effects on myocardial contraction and cardiac function using the living left heart model
Source: Front Bioeng Biotechnol. 2026 Apr 10;14:1756525. doi: 10.3389/fbioe.2026.1756525 (PMC13106318; doi:10.3389/fbioe.2026.1756525)
Supplement: Supplementary file 2 [file Table1.docx]

| **Strain type** | **Region** | **Slope (**$\boldsymbol{d}\left( \boldsymbol{APS} \right)\boldsymbol{/d}\boldsymbol{L}_{\boldsymbol{r}}$**)** | $\boldsymbol{R}^{\boldsymbol{2}}$ |
| --- | --- | --- | --- |
| Radial | Basal | +1.02 | 0.996 |
| Radial | Equatorial | +0.40 | 0.847 |
| Radial | Apical | +0.62 | 0.961 |
| Circumferential | Basal | −0.26 | 0.983 |
| Circumferential | Equatorial | −0.34 | 0.990 |
| Circumferential | Apical | −0.44 | 0.992 |
| Longitudinal | Septal | −0.10 | 0.781 |
| Longitudinal | Anterior | −0.10 | 0.781 |
| Longitudinal | Lateral | −0.06 | 0.750 |
| Longitudinal | Posterior | −0.10 | 0.781 |

**Table S1. Linear regression summary of APS versus** $\boldsymbol{L}_{\boldsymbol{r}}$**​ in the S0 series (S01–S04;** $\boldsymbol{L}_{\boldsymbol{0}}$**​ fixed)**

**Table S2. Piecewise linear regression summary of APS versus** $\boldsymbol{L}_{\boldsymbol{0}}$ **​(S11–S14;** $\boldsymbol{L}_{\boldsymbol{r}}$**fixed)**

low segment: S11–S12–S00 ($L_{0}$=1.48→1.53→1.58 μm)
High segment: S00–S13–S14 ($L_{0}$=1.58→1.63→1.68 μm)

| **Strain type** | **Region** | **Low slope (**$\boldsymbol{d}\left( \boldsymbol{APS} \right)\boldsymbol{/d}\boldsymbol{L}_{\boldsymbol{0}}$**)** | $\boldsymbol{R}^{\boldsymbol{2}}$ | **High slope (**$\boldsymbol{d}\left( \boldsymbol{APS} \right)\boldsymbol{/d}\boldsymbol{L}_{\boldsymbol{0}}$**)** | $\boldsymbol{R}^{\boldsymbol{2}}$ |
| --- | --- | --- | --- | --- | --- |
| Radial | Basal | −1.30 | 0.750 | −1.30 | 0.998 |
| Radial | Equatorial | +5.90 | 0.925 | −0.80 | 1.000 |
| Radial | Apical | +3.90 | 0.974 | −1.00 | 1.000 |
| Circumferential | Basal | −0.60 | 0.871 | +0.30 | 0.964 |
| Circumferential | Equatorial | −1.40 | 0.855 | +0.40 | 1.000 |
| Circumferential | Apical | −1.10 | 0.936 | +0.50 | 0.987 |
| Longitudinal | Septal | −0.90 | 0.907 | +0.20 | 1.000 |
| Longitudinal | Anterior | −0.70 | 0.750 | +0.20 | 1.000 |
| Longitudinal | Lateral | −0.80 | 0.923 | +0.20 | 1.000 |
| Longitudinal | Posterior | −0.70 | 0.993 | +0.30 | 0.964 |

**Table S3. Piecewise linear regression summary of APS along the** $\boldsymbol{L}_{\boldsymbol{r}}\boldsymbol{-}\boldsymbol{L}_{\boldsymbol{0}}$**​ (S21-S24)**

low segment: S21–S22–S00 ($L_{r}$=1.75→1.80→1.85 μm, $L_{0}$=1.48→1.53→1.58 μm)
high segment: S00–S23–S24 ($L_{r}$=1.85→1.90→1.95 μm, $L_{0}$=1.58→1.63→1.68 μm)

| **Strain type** | **Region** | **Low slope (**$\boldsymbol{d}\left( \boldsymbol{APS} \right)\boldsymbol{/d}\boldsymbol{L}_{\boldsymbol{r}}$**)** | $\boldsymbol{R}^{\boldsymbol{2}}$ | **High slope (**$\boldsymbol{d}\left( \boldsymbol{APS} \right)\boldsymbol{/d}\boldsymbol{L}_{\boldsymbol{r}}$**)** | $\boldsymbol{R}^{\boldsymbol{2}}$ |
| --- | --- | --- | --- | --- | --- |
| Radial | Basal | −0.20 | 1.000 | −0.40 | 1.000 |
| Radial | Equatorial | +6.30 | 0.957 | −0.50 | 0.987 |
| Radial | Apical | +4.30 | 0.991 | −0.30 | 0.964 |
| Circumferential | Basal | −0.80 | 0.842 | +0.10 | 0.750 |
| Circumferential | Equatorial | −1.80 | 0.984 | +0.10 | 0.750 |
| Circumferential | Apical | −1.30 | 0.983 | +0.10 | 0.750 |
| Longitudinal | Septal | −1.10 | 0.936 | +0.20 | 1.000 |
| Longitudinal | Anterior | −0.90 | 0.996 | +0.10 | 0.750 |
| Longitudinal | Lateral | −1.00 | 0.987 | +0.10 | 0.750 |
| Longitudinal | Posterior | −1.00 | 0.987 | +0.10 | 0.750 |
